# Repurposing a Small Molecule Plant Hormone as a Tunable ON‐Switch for CAR‐T Cell Immunotherapy

**DOI:** 10.1002/advs.77020

**Published:** 2026-08-11

**Authors:** Hongxiang Zeng, Lei Zheng, Rongkun Tao, Sirui Zheng, Yanke Zhong, Yongxia Niu, Peng Zhang, Xiang Ma, Chen Yang, Min Zhang, Fuyu Duan, Jing Yang, Xuan Wei, Xiang Zhou, Zhenbiao Yang, Zhaoxi Sun, Qizhou Lian

**Affiliations:** ^1^ Faculty of Synthetic Biology and Institute of Cell and Gene Technology Shenzhen University of Advanced Technology Shenzhen China; ^2^ State Key Laboratory of Quantitative Synthetic Biology Shenzhen Institute of Synthetic Biology Shenzhen Institutes of Advanced Technology Chinese Academy of Sciences Shenzhen China; ^3^ Department of Chemistry New York University New York USA; ^4^ New York University–East China Normal University Center for Computational Chemistry School of Chemistry and Molecular Engineering New York University Shanghai Shanghai China; ^5^ The First Affiliated Hospital of Xiamen University School of Medicine Clinical Research Institute Xiamen University Xiamen China; ^6^ Institute of Emerging Agricultural Technology Shenzhen University of Advanced Technology Shenzhen China; ^7^ Center For Translational Stem Cell Biology Hong Kong and State Key Laboratory of Pharmaceutical Biotechnology The University of Hong Kong Hong Kong SAR China; ^8^ Prenatal Diagnostic Center and Cord Blood Bank Guangzhou Women and Children's Medical Centre Guangzhou Medical University Guangzhou China

**Keywords:** auxin, cell biology, chimeric antigen receptor, cytotoxicity, hormone, immunogenicity, immunotherapy, plant hormone, small molecule, t cell

## Abstract

Precise regulation of chimeric antigen receptor (CAR)‐T cell activity is essential to maximize efficacy and minimize toxicity. While switch‐controlled CAR‐T holds great promise, challenges remain in achieving accurate activation, reducing immunogenicity, and preventing off‐target effects. Here we present a novel inducible ON‐switch CAR design that leverages plant hormone signaling components to achieve controllable T cell activation. By engineering a receptor system integrating the plant auxin receptor AFB1 with its co‐receptor IAA7, we enable ligand‐dependent interactions triggered by the plant hormone auxins. This design allows rapid, reversible, and dose‐dependent T cell activation, resulting in potent cytotoxicity against B‐cell lymphoma in vitro and in vivo. Notably, auxCAR‐T cells controlled by synthetic auxin analogs maintain a favorable memory phenotype and exhibit reduced functional exhaustion during treatment, leading to improved therapeutic efficacy. Our plant hormone‐based orthogonal system overcomes key limitations of existing switch systems and advances the development of precision CAR‐T therapies.

## Graphical abstract

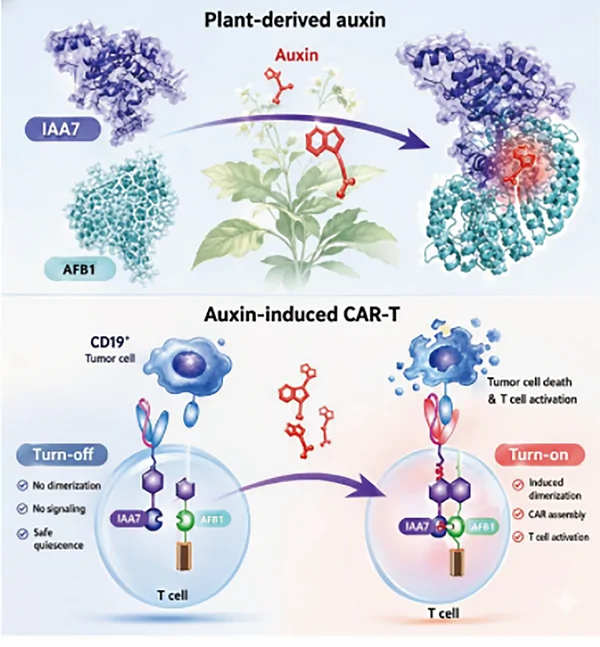

## 1 Introduction

Chimeric antigen receptor (CAR)‐T cell therapy has revolutionized cancer treatment, achieving remarkable remission rates in refractory blood cancers. However, its use is associated with severe adverse effects including cytokine release syndrome (CRS) [1, 2, 3, 4], neurotoxicity (ICANS) [5, 6, 7, 8], potentially fatal on‐target/off‐tumor effects [9], and CAR‐T functional exhaustion [9], which pose significant safety challenges and limit wider clinical adoption. These toxicities primarily result from uncontrolled CAR‐T cell activation [10, 11], highlighting the need for more precise and controllable regulation of CAR‐T activity. In response, several switchable CAR‐T (sCAR‐T) systems have been developed to enable reversible and targeted control of T cell activation.

Current switchable CAR‐T (sCAR‐T) systems primarily employ four switching mechanisms: chemical inducible dimerizers (CID) (e.g., rapamycin analogs) [12, 13, 14, 15, 16, 17], antibody‐based systems (e.g., FITC‐tagged CARs) [18, 19], optogenetic switches (e.g., LOV2‐ssrA/sspB or CRY2/CIBN pair) [20, 21], and protease‐activated CARs [22, 23, 24]. Members of the above sCAR‐T systems have been examined in preclinical and clinical stages such as rapamycin [15, 16], retinol binding protein 4 (hRBP4) [25], Venetoclax [26], lenalidomide‐responsive zinc finger scaffolds [27], and synthetic antibodies [28], offering improved specificity and reduced background activity. Recently, we also introduced a light‐inducible CAR system (LiCAR) capable of real‐time, spatially controlled T cell activation using upconversion nanomaterials [21]. Although the above sCAR‐T systems offer distinct advantages such as modularity, dose‐titration, spatiotemporal control, or tumor microenvironment responsiveness, they nevertheless still face critical limitations, including immunogenicity [29], off‐target effects, invasive activation requirements [30, 31, 32], or inconsistent enzymatic cleavage [22, 24]. Of note, despite clinical promise, no sCAR‐T therapy has been approved, as FDA concerns persist regarding toxicity control (CRS/neurotoxicity), switch reliability (unpredictable PK/immunogenicity), and durability (tumor escape). For instance, chemically inducible systems can suffer off‐target effects, incomplete activation, and reversibility issues. The FDA demands robust dosedependent control and fail‐safe mechanisms (e.g., suicide genes) to address these risks before approval [33]. Recent clinical holds on Bellicum's BPX‐601 (rapamycin analog) over cytokine toxicity highlight these challenges [34, 35]. Obviously, there is an urgent need to develop more robust, versatile, tunable, and clinically feasible switch systems suitable for the complex tumor environment.

Auxin is a naturally occurring plant hormone that plays a vital role in regulating various aspects of plant growth and development such as cell elongation, tissue differentiation, and organ formation. Mechanistically, indole‐3‐acetic acid (IAA), the primary auxin hormone, acts as a glue by binding to Auxin F‐Box receptors (TIR1 and AFB1‐AFB5) and their co‐receptors, Auxin/IAA repressor proteins (AUX/IAA, e.g., IAA7), and stabilizing the interaction between the receptors and co‐receptors. This interaction triggers the degradation of AUX/IAAs, releasing their repressive effects on Auxin Response Factors (ARFs) to activate the expression of auxin‐induced genes [36, 37]. The widespread presence of auxin in a variety of foods and plants highlights its natural abundance and biocompatibility. Importantly, extensive studies have shown that it is safe for human consumption, exhibiting minimal toxicity and side effects [38, 39, 40]. It is reported that IAA‐rich products derived from microbes or plants, such as yoghurt, probiotics, microgreens, and fresh carrots, introduced into the diet may be promising for disease management [41]. Of note, auxin and its receptors are absent in humans, conferring orthogonality and minimizing off‐target interactions. Furthermore, auxin exhibits favorable safety profiles and widespread presence in the human diet [38, 39, 40]. This favorable safety profile, combined with its specific biological functions in plants, makes auxin an attractive candidate for developing safe and effective switch systems in human cell therapies.

In this study, we engineered an innovative auxin‐inducible ON‐switch for CAR‐T cell therapy, called auxCAR, that integrated the plant receptor AFB1 and its co‐receptor IAA7. This design enabled their interactions specifically in response to auxin in a dosage‐dependent manner, allowing controlled and tunable activation of CAR‐T cells. Our findings demonstrate that auxCAR induced sensitive, rapid, dose‐dependent T cell activation, exhibited significant cytotoxicity against Raji B‐cell lymphoma in vitro, and increased resistance to exhaustion compared with conventional CAR‐T cells, and achieved potent anti‐tumor activity in vivo in an immunodeficient mouse model of hematolymphoma. AuxCAR‐T cells exhibited comparable antitumor activity to stCAR‐T cells, with a trend toward improved durability in survival and tumor burden control, and hold additional benefits including preserved naïve and memory T cells and reduced CAR‐T functional exhaustion in tumor microenvironments. Taken together, our results indicate that the auxin‐inducible auxCAR system is a promising platform for enhancing the safety, controllability, and effectiveness of CAR‐T cell therapies.

## 2 Results

### 2.1 Computational Prediction and Systematic Screening of IAA and AUX Pairs Identified Optimal Auxin‐Inducible Protein Dimerization

To engineer a chemically controllable chimeric antigen receptor (CAR) system (Figure 1A), we took advantage of the auxin‐inducible interaction between its receptor, the TIR1/AFB family proteins (e.g., AFB2, AFB5), and their co‐receptors, the AUX/IAA proteins (e.g., IAA5, IAA7, and IAA8) [37, 39, 40, 42, 43, 44]. TIR1/AFB members (TIR1/AFB1‐AFB5) exhibit differential localization to the nucleus or the cytoplasm. Given CAR components operating in the cytoplasm or the plasma membrane, we chose AFB1, which is naturally localized in the cytoplasm. In addition, AFB1 lacks the E3 ubiquitin ligase binding function [45], making it an attractive candidate for a non‐degradative and potentially auxin‐inducible CAR switch [46].

**FIGURE 1:**
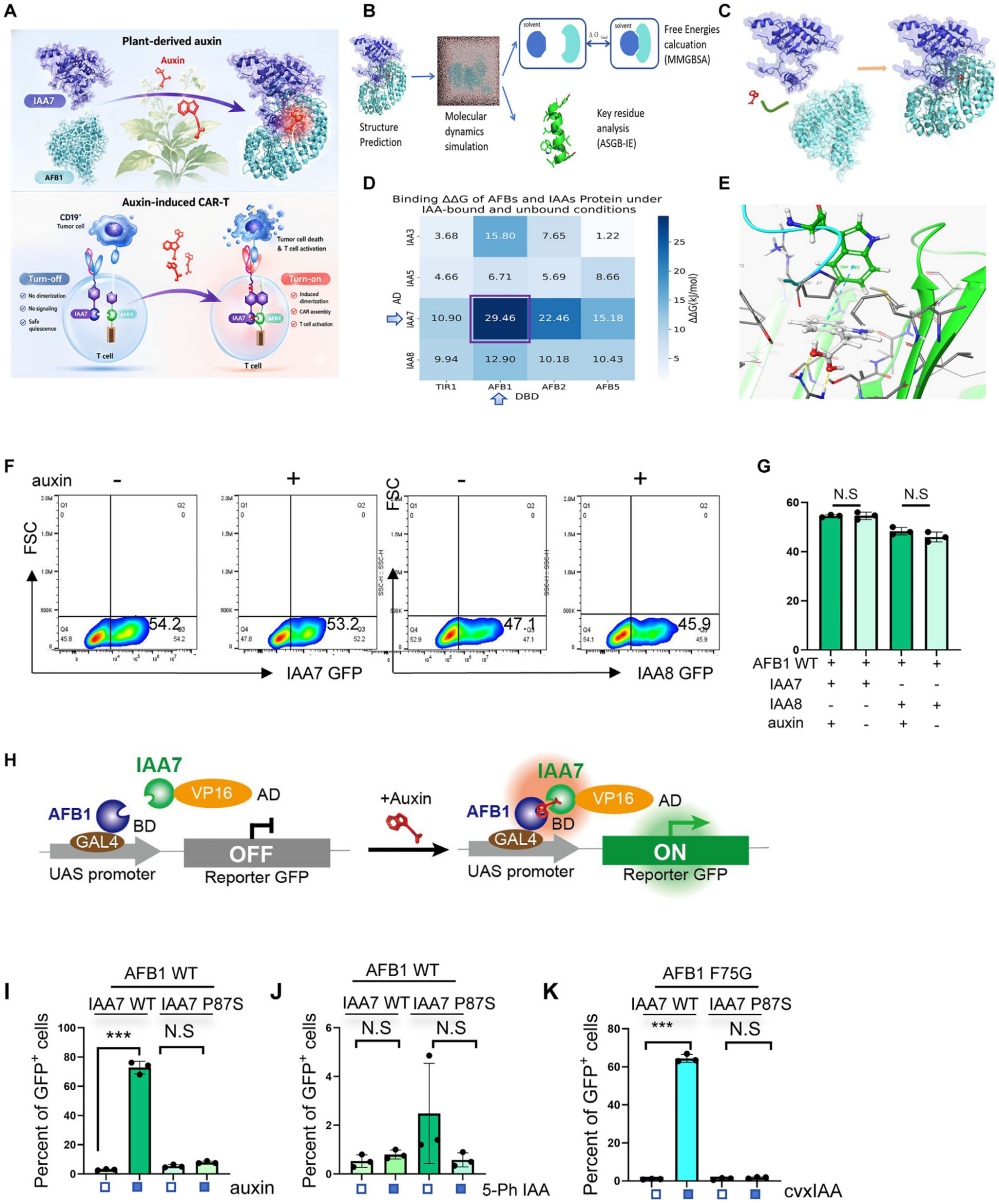
Screening of IAA and AFB Pairs for Auxin‐Inducible Protein Dimerization. (A) Design of synthetic CAR platforms with AND‐gate logic mediated by auxin sensing. (B) Workflow of computational research of AFBs and IAAs. (C) AFB1‐IAA7‐auxin ternary binding graph. (D) The heatmap of binding ∆∆G of AFBS protein and IAAs protein in the presence and absence of auxin. (E) The TRP85 in the DII domain forms a *π–π* stack (blue dotted line) with IAA that contributes to stronger binding. (F,G) Flow cytometry analysis (F) and statistical analysis (G) of GFP expression in HEK293T cells co‐transfected with WT AFB1 and IAA7‐GFP/IAA8‐GFP, under both auxin‐treated and auxin‐free conditions. (H) Schematic illustration of GFP reporter expression assay demonstrating auxin‐induced interaction between IAA7‐AD and AFB1‐BD fusion proteins. (I–K) Statistical analysis revealed that auxin (1 µM) (I), 5‐Ph‐IAA (1 µM) (J), and cvxIAA (1 µM) (K) induced GFP reporter expression in HEK293T cells co‐expressing AFB1 WT/ AFB1 F75G‐BD and IAA7 WT/IAA7 P87S ‐AD.

We employed several state‐of‐the‐art 3D complex prediction tools, including AlphaFold3, Proteinix, Chai‐1, and Boltz‐1, to model the structures of AFB1 in complex with IAA proteins (IAA3, IAA5, IAA7, and IAA8) and auxin (IAA) (Figure 1B) to perform theoretical calculations that complemented the experimental results presented in the referenced study (Figure S1A).

Initially, the predicted ternary complexes consistently exhibited an implausible structural feature: the extended N‐terminal regions of the IAA proteins penetrated the ring‐shaped structure formed by AFB1 (Figure 1C), contrary to known structural biology principles. Upon further investigation, we found that all available crystal structures included phytic acid as a placeholder molecule that significantly alleviated the observed artifact when incorporated into the predictions. This suggested that phytic acid (in plant cells) or its analog, inositol (in animal cells), may play a crucial role in stabilizing the assembly of the complex, potentially pointing to a novel direction for future research. Ultimately, we selected the AlphaFold3‐predicted structures as templates due to their superior accuracy. Due to the excessively long loop regions in the predicted crystal structures, we trimmed these regions to improve the accuracy of molecular dynamics simulations. Specifically, we retained 20 amino acids beyond the interface on both sides of the binding region while removing the excess loop segments. The following residues were deleted: residues 1–53 from IAA3, 1–47 from IAA5, 1–60 from IAA7, and 1–150 from IAA8. In the molecular dynamics simulations, the largest changes in binding free energy upon IAA binding occurred in the AFB1_IAA7 complex, with increases of 29.46 kJ/mol (Figure 1D). These results indicated that auxin had the strongest activating effect in this combination. The DII domain (GWPPV motif) is considered the key region mediating the interaction between AFBs and AUX/IAAs [47]. In all known crystal structures (e.g., PDB ID: 2P1P), AUX/IAAs are bound near the DII domain. In our analysis of key residues, TRP85 showed the largest change in binding free energy, an increase of 2.33 kJ/ in the presence of IAA (Figure 1E). The Trp85 in IAA7 made *π–π* stacking interactions directly atop the auxin molecule, anchoring the peptide in the receptor pocket. Mutation of this Trp to serine abolished Aux/IAA binding, underscoring its critical role and confirming our prediction (Figure S1B).

To experimentally validate the results of molecular dynamics simulations, we co‐transfected GFP‐tagged IAA7 and wild‐type AFB1 into HEK293T cells and confirmed that AFB1 did not mediate the degradation of IAA7 or IAA8, even in the presence of auxin (Figure 1F,G). We then assessed the dimerization between AFB1 and IAA7 using the split transcriptional activation assay (Figure 1H). This system produced robust GFP reporter activation only under auxin induction, and not in its absence, confirming efficient and specific dimerization. To further investigate the specificity of the interaction, we mutated a critical residue (P87S) within Domain II of IAA7, responsible for AFB binding [48]. This mutation abolished auxin‐induced GFP expression, confirming the essential role of this residue in auxin‐mediated dimerization (Figure 1I and Figure S2A). Next, we employed and evaluated the ligand specificity using 5‐Ph‐IAA, another synthetic auxin analog [49, 50, 51, 52, 53]. Unlike natural auxin, 5‐Ph‐IAA failed to induce GFP expression in cells co‐expressing IAA7 and AFB1, indicating that the IAA7–AFB1 interaction was highly specific to natural auxin (Figure 1J and Figure S2B). Furthermore, a mutation of phenylalanine (F) at position 75 to glycine (G) was introduced to AFB1. This AFB1 F75G mutation confers on the receptor the ability to specifically respond to another synthetic auxin analog, cvxIAA. cvxIAA efficiently induced the activation of the AFB1 F75G mutant, comparable to auxin‐induced activation of wild‐type AFB1 in the Jurkat NFAT‐GFP model (Figure 1K and Figure S2C).

In summary, our findings establish the IAA7‐AFB1 pair as an optimal module for the design of split auxin‐inducible CARs. This pair offers strong auxin‐dependent activation, minimal background activity, and precise ligand specificity, making it a promising candidate for safe and effective therapeutic application.

### 2.2 Design and Engineering of an Auxin‐Inducible CAR System With IAA7‐AFB1 Pair

We proposed a split chimeric antigen receptor (CAR) system, where the receptor is functionally divided into two components: the R chain and the S chain. These components were fused with the AFB1 and IAA7 domains to enable activation of the CAR only upon dimerization triggered by the presence of auxin.

We created an auxin‐inducible CAR, abbreviated as auxCAR, by splitting the CD19‐CAR tumor antigen‐binding receptor chain (R) and the signal transduction (S) chain and recombining them with IAA7 and AFB1, respectively (Figure 2A). The IAA7‐R chain consisted of a CD8 secretion signal peptide, an anti‐CD19 single‐chain variable fragment (scFv: FMC63), the hinge and transmembrane (TMD) domains of CD8α, the 41BB intracellular domain for co‐stimulation, IAA7, and a c‐Myc tag. For the AFB1‐S chain, we included a nucleus‐excluded signal (NES) peptide at the N‐terminus, followed by a homodimeric DAP10 scaffold protein [54] that was linked to the hinge and transmembrane (TMD) domains of CD8α, the AFB1 domain, flanked by the intracellular phosphorylation domain 41BB and CD3ζ. The C‐terminal CD90.1 (Thy1.1) was co‐expressed with CD3ζ, separated by a P2A peptide, for sorting and enrichment of CAR‐positive T cells following lentiviral transduction (Figure 2A).

**FIGURE 2:**
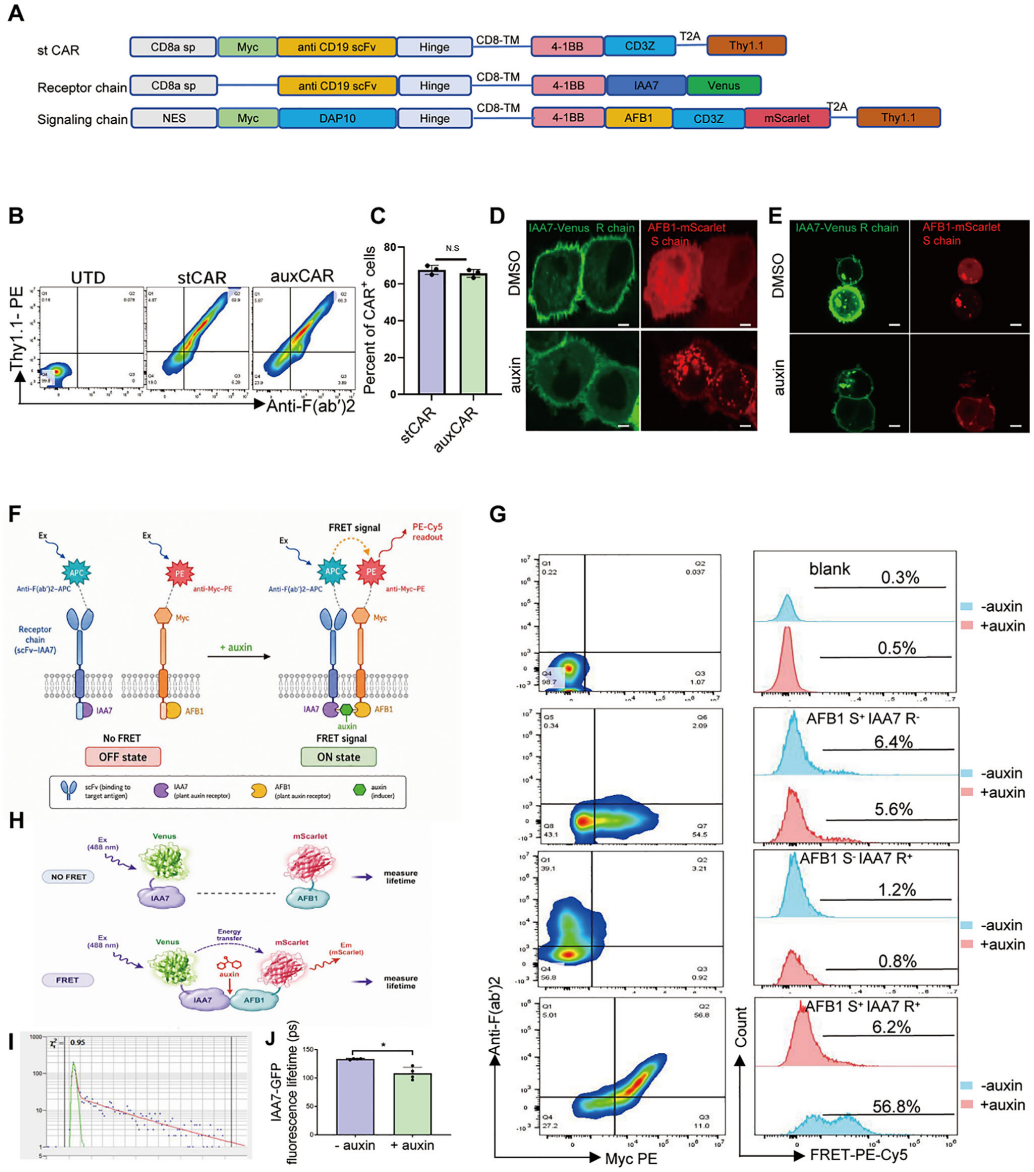
Design of an auxin‐inducible CAR system with IAA7/AFB1 pair. (A) Architecture of the aux‐CAR. Upper: cartoon depicting the different components. Bottom: schematic of the R‐ and S‐chains encoded in a single lentiviral vector, each separated by Venus or mScarlet. (B,C) Flow cytometry evaluation (B) and Statistical (C) analysis of IAA‐R‐chain and AFB1‐S‐chain expression in Jurkat cells detected by anti‐mouse F(ab’) Ab‐APC (binds to the anti‐CD19 scFv) and anti‐mouse Thy1.1‐PE, respectively. The untransduced (UTD) cells were designated as the blank control. Three technical replicates were performed with similar results. (D,E) Representative confocal images showing the reversible recruitment of the cytosolic construct (AFB1‐S chain‐mScarlet tagged, red) toward the PM‐resident construct A (IAA7‐R chain Venus tagged, green) in response to auxin induction in HeLa (D) and Jurkat (E) cells. Scale bar, 5 µm. (F) Schematic illustration of FRET detection following dual antibody staining and auxin‐mediated IAA7 R chain and AFB1 S chain dimerization. (G) Representative Flow cytometry analysis revealed AFB1 S^+^ IAA7 R^−^, AFB1 S^−^ IAA7 R^+^, and AFB1 S^+^ IAA7 R^+^ expression with or without auxin induction. Detection of the PE‐Cy5 FRET signal ONLY within the dual IAA R^+^/AFB1 S^+^ group. (H) Schematic illustration of fluorescence lifetime changes observed in auxin‐induced FRET FLIM. (I) A representative curve fit for the lifetime decay of IAA7 S chain‐Venus and AFB1 S chain‐mScarlet induction with auxin using a bi‐exponential fit. The calculated IRF (green curve) and overall decay pattern (red curve) are presented. (J) The average lifetime of Venus fluorescence determined for HeLa cells transiently transfected with IAA7 S chain‐Venus and AFB1 S chain‐mScarlet with or without auxin induction. Bars represent the mean (mean ± s.d.) lifetime measured for 6 individual cells over three biological replicates.

We co‐transduced Jurkat CD4^+^ human lymphoid leukemia cells with the IAA7‐R chain and AFB1‐S chain via lentiviral vectors. Following transduction, we assessed the surface expression of both the auxCAR and CD19‐CAR using flow cytometry. The results showed that the surface expression of the auxCAR in Jurkat cells was comparable to that of CD19‐CAR (Figure 2B,C), indicating successful expression of both CAR constructs.

To investigate the localization and spatial rearrangement of the IAA7‐R and AFB1‐S chains following auxin induction, we tagged the IAA7‐R chain with Venus, a GFP with improved chromophore formation and brightness [55], and the AFB1‐S chain with mScarlet, a bright monomeric red fluorescent protein for cellular imaging [56]. In the absence of auxin, Venus‐tagged IAA7‐R was localized at the plasma membrane of HeLa cells, while mScarlet‐tagged AFB1‐S was predominantly found in the cytoplasm. Upon addition of auxin for 30 min, confocal fluorescence microscopy revealed that mScarlet rapidly trafficked to the plasma membrane, co‐localizing with the Venus signal (Figure 2D). We also observed comparable effects in Jurkat cells co‐transduced with lentiviral constructs expressing the IAA7 receptor chain and AFB1 signaling chain (Figure 2E). This result indicated that auxin‐induced dimerization of IAA7‐R‐Venus and AFB1‐S‐mScarlet effectively brought the two components together on the membrane, providing a basis for T cell activation and signaling.

To further characterize auxin‐induced dimerization of the IAA7‐R and AFB1‐S chains, we measured fluorescence resonance energy transfer (FRET) by flow cytometry [57]. In this assay, we used PE‐labeled anti‐c‐Myc tag antibody as the donor fluorophore and APC‐labeled anti‐CD19 scFv antibody as the acceptor. FRET signals were detected in the PE‐Cy5 channel (Figure 2F) and confirmed that auxin‐induced dimerization occurred between the IAA7‐R and AFB1‐S chains (Figure 2G). This finding demonstrated that the presence of auxin triggered the formation of a heterodimeric complex between the two CAR components.

To achieve a more precise characterization of the dimerization process, we used fluorescence lifetime imaging microscopy (FLIM)‐based FRET [58]. This technique measured the fluorescence lifetime of GFP‐tagged IAA7 constructs under auxin induction. GFP, known for its bright and photostable fluorescence, was used to track the FRET signal that exhibits a single exponential decay. Time‐correlated single photon counting (TCSPC) was employed to measure FRET and fluorescence decay in fixed cells (Figure 2H). FLIM images were captured to analyze the fluorescence lifetime of GFP, providing insight into the changes in FRET efficiency. The FRET‐FLIM analysis showed decreased fluorescence lifetime for the IAA7‐R‐Venus in HeLa cells after auxin induction (Figure 2I,J). This further confirmed successful dimerization of the IAA7‐R chain and AFB1‐S chain in the presence of auxin.

In summary, we demonstrated the dimerization of the IAA7‐R chain and AFB1‐S chain in T cells after auxin induction using three independent methods: laser confocal microscopy, FRET‐FACS, and FRET‐FLIM. These results confirm the auxin‐induced assembly of the split CAR system and provide a solid foundation for further development of auxin‐inducible CAR‐T cell therapies.

### 2.3 Assessment of ON‐Switch Functions of auxCAR‐T Cells in Jurkat T Cells

To assess T cell activation, we expressed standard CAR (stCAR) and auxin‐switched CAR (auxCAR) into Jurkat T cells by lentivirus. CAR‐T cells required the presence of hCD19 antigen for activation, with consequent increased CD69 expression, an early activation marker. AuxCAR‐T cells showed no significant change in CD69 expression when co‐cultured with human CD19‐negative (hCD19^−^) K562 leukemia cells or Raji lymphoma cells expressing the hCD19 antigen in the absence of auxin. In the presence of both the cognate tumor antigen (hCD19^+^) and auxin, auxCAR‐T cells exhibited a significant auxin‐dependent increase in CD69 expression (Figure 3A,B). Importantly, it showed that the responsive activation of CAR‐T to auxin was very sensitive and dose‐dependent. In the absence of auxin, CAR‐T activation was almost undetectable (less than 5%). At a level of auxin as low as 1 µM, almost 60% of T cells were activated (Figure 3B), indicating their sensitivity to auxin. These results confirmed successful construction of the ON‐switch auxCAR and demonstrated that auxin can be used to regulate T cell activation.

**FIGURE 3:**
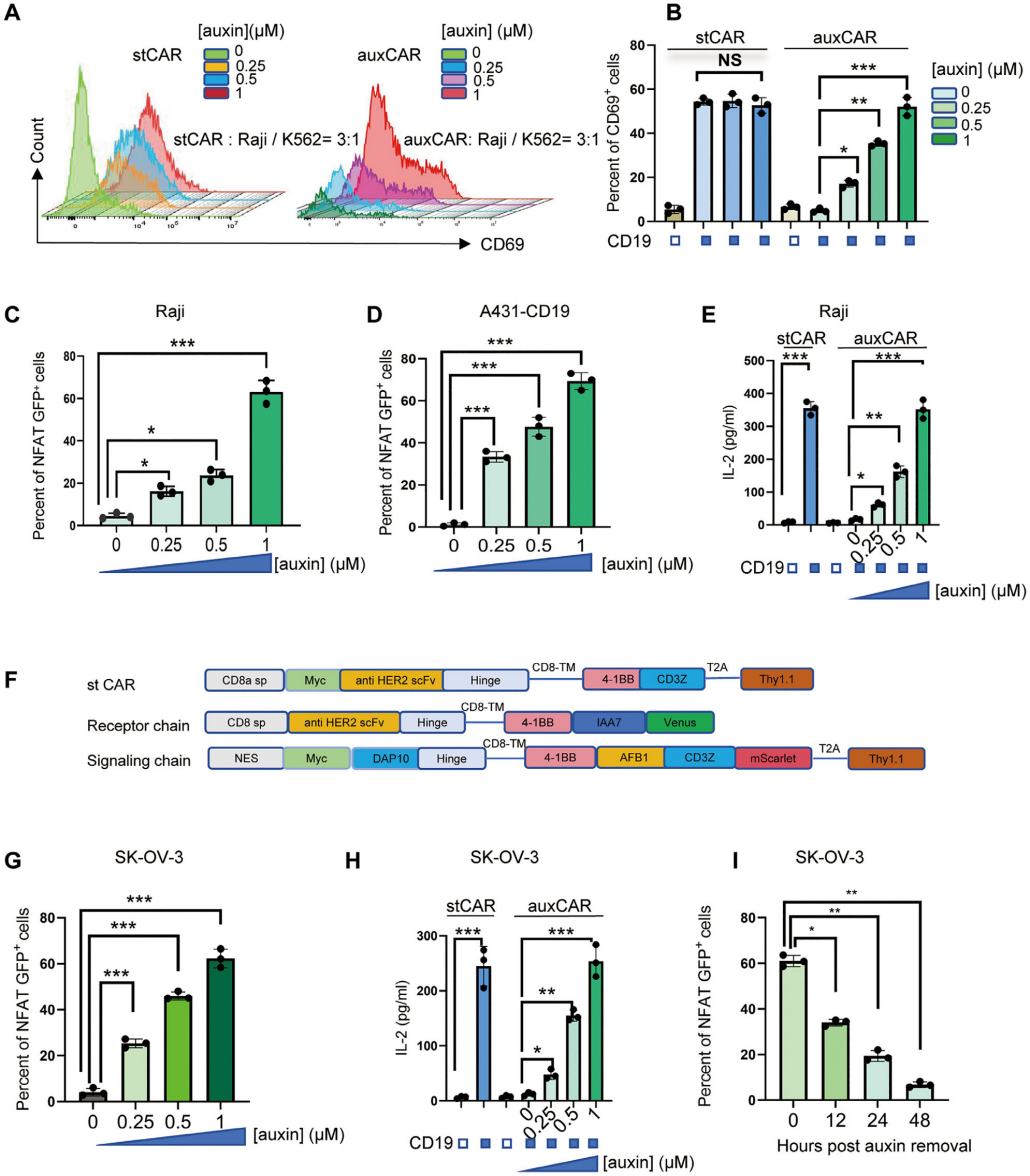
Assessment of auxCAR‐T cell performance in Jurkat T cells. (A,B) Representative Flow cytometry (A) and Statistical quantification (B) of early T cell activation as reflected by upregulated CD69 expression on the cell surface. Jurkat T cells transduced with stCAR (left) and auxCAR (right) were co‐cultured with hCD19^+^ Raji cells or hCD19^−^ K562 cells, with or without auxin. n = 3 independent biological replicates (mean ± s.d.). The *p*‐values were calculated using one‐way analysis of variance (ANOVA). (C,D) Statistical quantification of NFAT‐GFP reporter activity in Jurkat T cells expressing stCAR and auxCAR co‐cultured with tumor cells with hCD19^+^ Raji cells (C) or A431‐CD19 (D) with the indicated auxin concentration. n = 3 independent technical replicates (mean ± s.d.). The *p*‐values were calculated using one‐way analysis of variance (ANOVA). (E) Auxin‐dose‐dependent responses of stCAR T and auxCAR‐T cells examined by IL‐2 production in Jurkat T cells. (F) Architecture of the auxCAR HER2. Upper: cartoon depicting the different components. Bottom: schematic of the R‐ and S‐chains encoded in a single lentiviral vector, each separated by Venus or mScarlet. (G,H) Statistical quantification of NFAT‐GFP reporter activity (G) and IL‐2 production (H) in Jurkat T cells expressing stCAR HER2 or auxCAR HER2 co‐cultured with SK‐OV‐3 tumor cells in the presence of the indicated auxin concentration. n = 3 independent technical replicates (mean ± s.d.). (I) Statistical quantification of NFAT‐GFP reporter activity at the indicated time points following auxin removal in Jurkat T cells expressing auxCAR and co‐cultured with SK‐OV‐3 tumor cells. The auxCAR T cells were induced with 1 µM auxin.

To further investigate T cell activation under auxin induction, we used an NFAT‐driven GFP reporter gene to monitor the activation process. Jurkat T cells expressing the engineered auxCAR and co‐cultured with Raji lymphoma cells (with endogenously expressed human CD19 antigen) (Figure 3C and Figure S4A) or A431‐CD19^+^ solid tumor cells (with exogenous human CD19 antigen overexpression) (Figure 3D and Figure S4B) showed a dose‐dependent increase in GFP expression, indicating auxin‐dependent activation of the engineered T cells. The increase in GFP expression correlated with auxin concentration, suggesting that the degree of T cell activation could be finely tuned by adjusting auxin level. To assess the reversibility of auxCAR‐T cell activation, we evaluated the speed of T cell inactivation following auxin withdrawal. After removing auxin in co‐culture with CD19‐positive target tumor cells, a decrease in GFP production to baseline level was observed within 24 h, indicating that the T cells were effectively inactivated post‐auxin removal. Notably, after this brief inactivation period, auxCAR Jurkat NFAT GFP cells could be reactivated upon the reintroduction of auxin and subsequent co‐culture with CD19‐positive target tumor cells (Figure S4C). This demonstrated the reversible nature of auxCAR‐T cell activation, providing flexibility for precise control over T cell activity. Moreover, enzyme‐linked immunosorbent assay (ELISA) measurements revealed that auxCAR‐T cells significantly increased their IL‐2 secretion (Figure 3E). Following NFAT translocation to the cell nucleus. This further confirmed that auxin induction enabled precise regulation of T cell activation through the auxCAR system.

To assess the potential impact of auxin on the immune function of auxCAR‐T cells, we performed RNA transcriptome sequencing on primary human CD8^+^ T cells transduced with either auxCAR or stCAR. RNA‐seq analysis revealed no significant differential expression of immune function‐related genes between the two groups (Figure S3A,B), suggesting that the introduction of auxCAR components did not substantially alter the immune profile of T cells. Auxin or its analog cvx‐IAA does not cause baseline activation of T cells, as evidenced by almost no increase in CD69 expression (Figure S3C) and minimal impact on T cell proliferation (Figure S3D). These data support the notion that auxCAR can be used as a safe, controllable switch to regulate CAR‐T cell activity in vivo.

In order to show the power of the auxin‐induced IAA‐AFB1 system, we developed an auxin‐inducible chimeric antigen receptor (CAR) directed against the HER2 antigen, which is highly expressed on solid tumors (Figure 3F). We observed auxin dose‐dependent NFAT‐GFP expression (Figure 3G and Figure S4D) and IL‐2 secretion (Figure 3H). When auxCAR HER2 Jurkat T cells were co‐cultured with HER2‐positive SK‐OV‐3 tumor cells, this was comparable to that of stCAR HER2 Jurkat T cells, demonstrating the robustness of the auxin‐inducible system.

To enable signal reduction over 48 h following inducer withdrawal, we employed an NFAT‐driven GFP reporter system to monitor reversibility. Jurkat T cells expressing the engineered auxCAR and NFAT‐GFP reporter were co‐cultured with HER2‐positive SK‐OV‐3 tumor cells. Upon auxin washout, we observed gradual signal reduction over 48 h following inducer withdrawal. (Figure 3I and Figure S4E). These data demonstrate that the system is tightly controllable, consistent with the reversibility kinetics observed in this Jurkat NFAT‐GFP reporter washout experiment. While the system is reversible, the 48 h timescale may not be suitable for acute CRS intervention.

Taken together, these results demonstrated the regulation of T cell activation and inactivation using the auxCAR system. Auxin‐induced activation, measured by CD69 expression, NFAT‐driven GFP expression, and IL‐2 secretion, was dose‐dependent and reversible, highlighting the potential of the auxCAR system for controlled T cell therapies.

### 2.4 In Vitro Functions of AuxCAR T Cells Against hCD19 and HER2 Expressing Hematologic and Solid Tumors

We first evaluated the ability of auxCAR‐transduced primary peripheral blood CD8^+^ T cells to form immune synapses in vitro with both hematological and solid tumors expressing the CD19 antigen. A hallmark of primary CAR‐T cells is their capacity to form immune synapses with target tumor cells, leading to the release of granules and perforins that induce apoptosis in the target cells. Controlling CAR‐T cell activation to trigger cytotoxicity against tumors is a crucial feature of “smart” immunotherapy.

To determine whether auxCAR could regulate T cell activation, we co‐transduced peripheral blood CD8^+^ T lymphocytes with the IAA7‐R chain and AFB1‐S chain using lentiviral vectors. We then assessed the surface expression of CD19 scFv and Thy1.1 by flow cytometry. The results demonstrated that the expression level of st CAR and auxCAR on primary human CD8^+^ T lymphocytes was comparable (Figure 4A,B).

**FIGURE 4:**
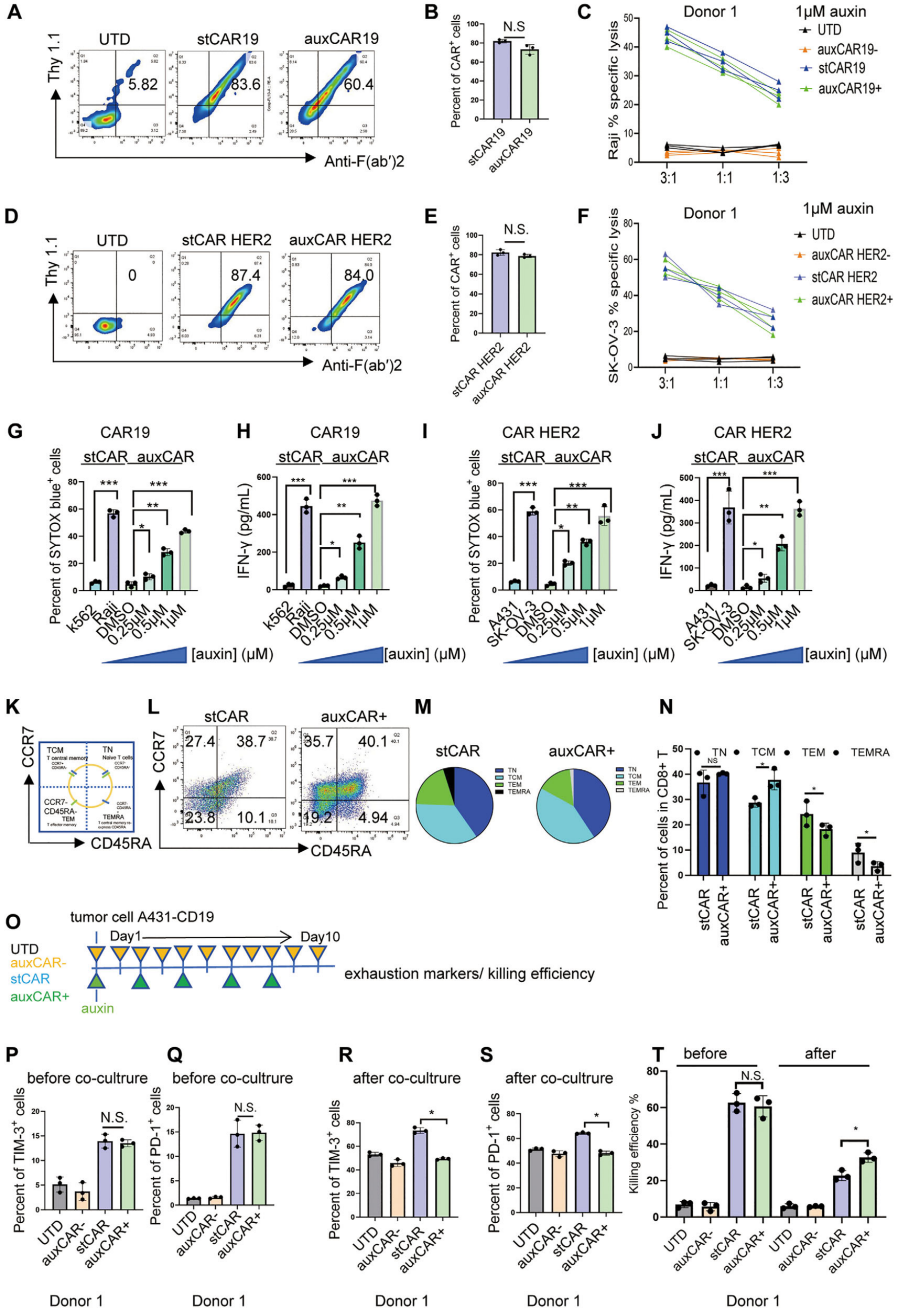
Estimation of the in vitro performance of AuxCAR‐transduced primary T cells against hematological and solid tumors. (A,B,D,E) Flow cytometry (A,D) and Statistical analysis (B,E) of evaluation of R‐ and S‐chain expression on primary human CD8^+^ T lymphocytes detected by anti‐mouse F(ab’) Ab‐APC, which binds the anti‐CD19 scFv (A,B) and anti‐HER2 scFv (C,D) and anti‐mouse Thy1.1‐PE, respectively. UTD (Untransduced) as a blank control. n = 3 n = 3 independent experiments (with 1 donor per experiment). (mean ± s.d.). (C,F) Cytotoxicity against target cells was evaluated at the indicated effector‐to‐tumor (E:T) ratios (3:1, 1:1, 1:3) using auxCAR19 T cells (C) or auxCAR19 HER2 T cells (F) from healthy donor 1 activated with 1 µM auxin. stCAR‐transduced T cells were used as a positive control in all experiments. n = 3 independent experiments (with 1 donor per experiment). (G–J) Statistical quantification of SYTOX blue‐positive cells to determine cytotoxicity (G,I) and Interferon‐gamma (IFNγ) production (H,J) in auxCAR‐19 T cells cocultured with Raji cells (G,H) and auxCAR‐HER2 T cells cocultured with SK‐OV‐3 cells (I,J), with a gradient concentration of auxin indicated. n = 3 independent experiments (with 1 donor per experiment). stCAR‐transduced T cells were used as a positive control in all experiments. (K) Schematic illustration of Subsets of T cells using CCR7 and CD45RA differentiation. (L–N) Representative Flow cytometry evaluation (L) and Pie charts (M) and statistical analysis of bar (N) charts of naive (TN: CD45RA^+^CCR7^+^), central memory (TCM: CD45RA^−^CCR7^+^), effector memory (TEM: CD45RA^−^CCR7^−^), and terminally differentiated effector memory (TEMRA: CD45RA^+^CCR7^−^) T cells in auxCAR and stCAR‐T by CCR7 and CD45RA staining. n = 3 independent experiments (with 1 donor per experiment). (mean ± s.d.). The *p*‐values were calculated using the two‐sided unpaired Student's *t*‐test. (O) Schematic illustration of repeated tumor stimulation assay. CAR‐T cells were restimulated with A431‐CD19 tumor cells at a 1:1 E:T ratio for 10 days. Auxin was refilled every 48 h. (Q–S) Statistical analysis of the expression level of exhaustion marker TIM‐3 (P,R), PD‐1 (Q,S) on CAR T cells before (P,Q) and after (R,S) exposure to the repeated tumor stimulation assay. (T) Cytotoxicity of the CAR T cells before (left) and after (right) co‐culture assay at an E:T ratio of 1:1. n = 3 independent experiments (with 1 donor per experiment).

To assess the tumor‐killing ability of the engineered T cells, we co‐cultured auxCAR primary CD8^+^ T cells with CD19‐positive target tumor cells. Cytotoxicity was measured using SYTOX blue staining (Figure S5A) and the production of IFN‐γ in the supernatant. The results showed a clear trend of auxin‐dependent enhanced tumor cell killing (Figure 4G and Figure S5B) and increased IFN‐γ production in the supernatant, with the efficiency of auxCAR‐T cells comparable to that of CD19‐CAR‐T cells. The killing efficiencies of both stCAR and auxCAR (induced with 1 µM auxin) were comparable across various effector‐to‐target ratios (Figure 4C). Notably, in the absence of auxin, both auxCAR and stCAR‐T cells showed low levels of target cell killing, though this leaky activity was minimal. Upon addition of auxin to the culture medium, there was a significant increase in SYTOX Blue staining (Figure 4G) and IFN‐γ secretion (Figure 4H) in Raji cells, which is comparable to the auxCAR‐T group, indicating robust cytotoxicity. Importantly, when co‐cultured with hCD19‐negative K562 cells, neither auxCAR nor CD19‐CAR T cells induced SYTOX Blue staining, confirming their specificity for CD19‐positive targets. The above ex vivo cytotoxicity results were further confirmed in T cells derived from two additional donors (Figure S5D,E).

We also demonstrated cell‐surface expression of the anti‐HER2 auxCAR and corresponding anti‐HER2 stCAR in CD8^+^ T lymphocytes (Figure 4D,E). The results showed a clear trend of auxin‐dependent enhanced HER2‐positive SK‐OV‐3 tumor cell killing (Figure 4I and Figure S5C) and increased IFN‐γ production (Figure 4J) in the supernatant, with the efficiency of auxCAR HER2 T cells comparable to that of HER2 CAR‐T cells. The killing efficiencies of both stCAR HER2 and auxCAR HER2 T cells (induced with 1 µM auxin) were comparable across various effector‐to‐target ratios (Figure 4F). Importantly, when co‐cultured with hCD19‐negative A431 cells, neither auxCAR HER2 nor stCAR HER2 T cells induced SYTOX Blue staining (Figure 4I), confirming their specificity for HER2‐positive targets.

In CAR‐T cell therapy, T cell subsets with early memory phenotypes are associated with enhanced proliferation, anti‐tumor activity, and persistence in vivo. To determine whether auxCAR affected the proportion of memory T cell subsets, we evaluated the distribution of naive (TN: CD45RA^+^CCR7^+^), central memory (TCM: CD45RA^−^CCR7^+^), effector memory (TEM: CD45RA^−^ CCR7^−^), and terminally differentiated effector memory (TEMRA: CD45RA^+^CCR7^−^) T cells (Figure 4K) in auxCAR and stCAR‐T cells. Consistent with our expectations, after activation, transduction, and expansion, auxCAR‐T cells retained a slightly higher frequency of central memory T cells (TCM) compared with stCAR‐T cells. The proportion of effector memory T cells (TEM) was similar between auxCAR and stCAR‐T cells (Figure 4L–N). These results suggest that auxCAR‐T cells maintained a beneficial central memory subset profile, associated with enhanced long‐term anti‐tumor efficacy.

We next determined whether auxCAR‐T cells were more resistant to exhaustion than stCAR‐T cells. We subjected both CAR‐T cell groups to repeated stimulation with homotypic tumor cells (A431‐CD19) in vitro (Figure 4O). At the resting state, both stCAR‐T and auxCAR‐T cells showed a similar low expression of exhaustion markers TIM‐3 and PD‐1 by flow cytometry (Figure S6A). Nonetheless, ten days following tumor, stCAR‐T cells showed a more significant increase in exhaustion markers, with a higher proportion of TIM‐3^+^ (Figure 4P,R and Figure S6C,E) and PD‐1^+^ cells (Figure 4Q,S and Figure S6D,F) than the auxCAR‐T group with auxin induction. These results suggested that auxCAR‐T cells may exhibit greater resistance to exhaustion, potentially improving their persistence and anti‐tumor activity in vivo. Furthermore, We also found that the killing capacity of auxin pre‐activated auxCAR T cells and stCAR T cells was comparable prior to chronic antigen stimulation, while the killing efficiency of auxCAR‐T cells against A431‐CD19 target cells was 35.5% ± 5%, which was significantly higher than that of stCAR‐T cells: 25.8% ± 5% (*p*< 0.05) after 4 round tumor cell repeated stimulation (Figure 4T and Figure S6B). The above ex vivo exhaustion (Figure S6G,H) and cytotoxicity (Figure S6I,J) results were further confirmed in T cells derived from two additional donors. These new data directly demonstrate that the favorable memory/low exhaustion phenotype translates into improved functional durability. This provides direct functional evidence that auxCAR‐T cells preserve effector capacity better upon chronic antigen exposure.

In summary, auxCAR‐T cells effectively induced cytotoxicity against CD19‐positive target cells in an auxin‐dependent manner, maintained a favorable memory T cell subset profile, and showed enhanced resistance to exhaustion compared with stCAR‐T cells. These results support the potential use of auxCAR as a safe switch to control CAR‐T cell activity in therapeutic settings and highlight the promise of the auxCAR system for more controlled and efficient CAR‐T cell therapies.

### 2.5 Primary Anti‐CD19 AuxCAR‐T Cells Effectively Destroyed B‐Cell Lymphoma in Vivo

To better simulate the clinical scenario of hematological tumor patients, we established a Raji‐induced B‐cell lymphoma model in mice by tail vein injection of CAR‐T cells. In vivo experiments were conducted to compare the efficacy of auxCAR‐T cells with stCAR‐T cells in inhibiting the progression of B‐cell lymphoma (Figure 5A). To exclude the possibility of baseline activation potentially arising from mouse gut microbiota or metabolism [59], we used this AFB1 F75G mutation (Figure 1K), which confers the receptor the ability to specifically respond to the synthetic auxin analog cvxIAA [46], a compound that is virtually absent in mice. The cvxIAA‐auxin/IAA‐AFB1 receptor pair is orthogonal, meaning it activates auxin signaling without interfering with endogenous auxin pathways or TIR1/AFBs. In vivo, cvxIAA effectively hijacked downstream auxin signaling, as evidenced by changes at the transcriptomic level and in specific developmental contexts [60]. To demonstrate that the AFB1 F75G variant functions analogously to the original AFB1, we performed an orthogonal functionality assay. We observed that AFB1 F75G exhibits potent cytotoxic activity exclusively upon induction with cvxIAA, while showing no response to natural auxin (Figure 5B).

**FIGURE 5:**
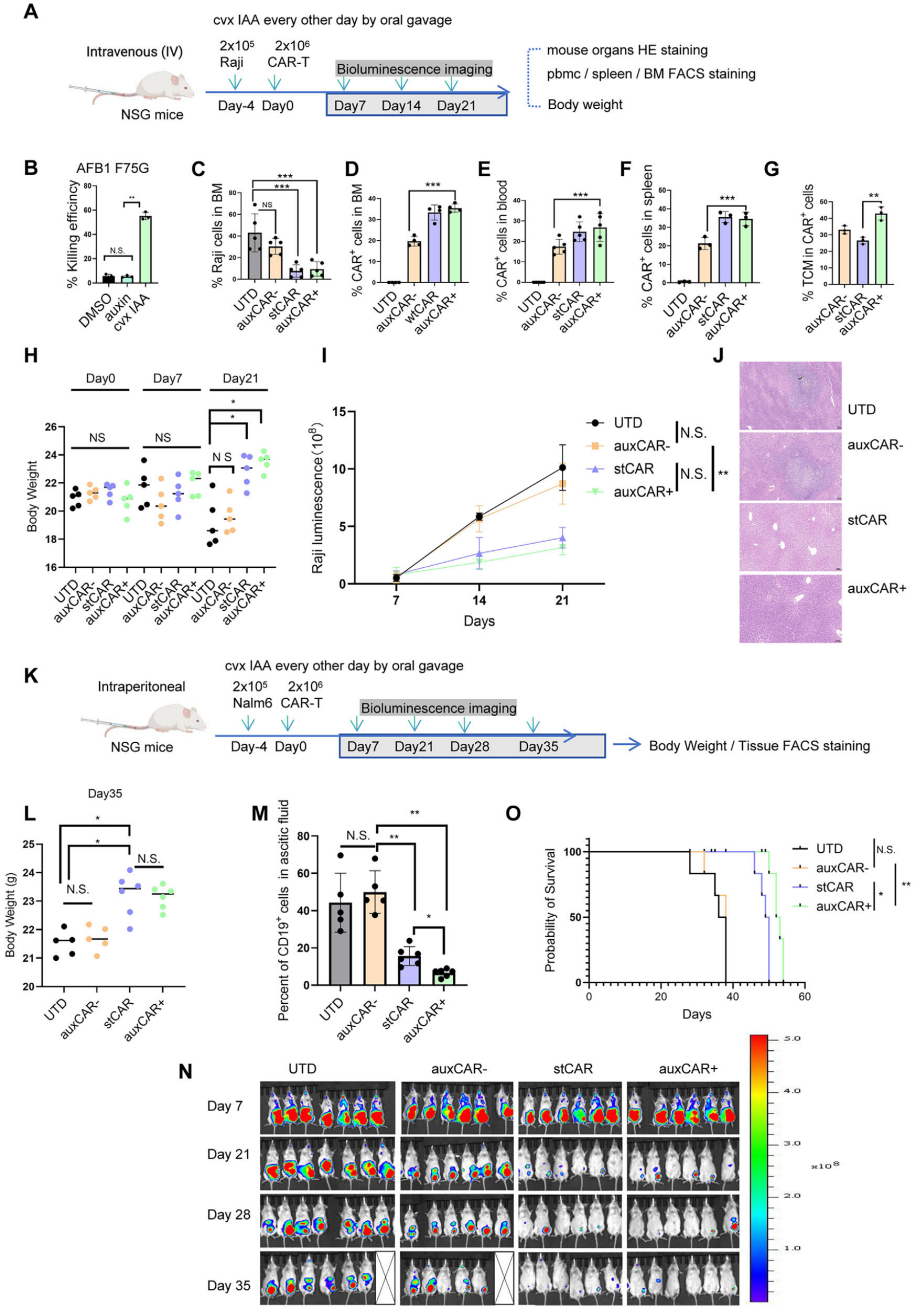
AuxCAR‐positive primary CD8^+^ T cells delayed Raji B cell lymphoma and Nalm6 intraperitoneal tumor burden model, in vivo. (A) Schematic of the experimental set‐up for systemic injection of stCAR and auxCAR T cells. First, 2 × 10^5^ firefly luciferase (FLuc)‐transduced Raji cells were injected into the tail vein (i.v.) of C‐NKG mice. After 4 days, 2 × 10^6^ UTD (Untransduced), stCAR T, or auxCAR T cells were systemically injected by tail vein injection (i.v.). The mice were subjected to cvxIAA (50 mg/kg) administration by oral gavage every other day. The auxin‐induced auxCAR T‐cell group was designated as auxCAR+, and the group not subjected to cvxIAA induction was designated as auxCAR‐. At day 21, mice were euthanized for organ isolation and phenotypic analyses. (B) Orthogonal functionality measured by in vitro CD8^+^ T cell killing efficiency of the AFB1‐F75G mutant confers the receptor the ability to specifically respond to the synthetic auxin analog cvxIAA but not to natural auxin (IAA), and (C–G) statistical analysis of CD19^+^ Raji tumor cells in the bone marrow (C) was determined on day 21 post‐injection. Statistical analysis of CAR^+^ T cell persistence in the bone marrow (D), blood (E), spleen (F), and central memory (TCM: CD45RA^−^CCR7^+^) cells (G) gated from CAR^+^ T cells was determined on day 14 post injection. (H) Statistical analysis of mouse body weights of mice measured on day 0, day 7, and day 21. Dots represent individual mice; bars represent mean ± s.d. Statistical significance among groups was determined using one‐way ANOVA, n = 5 mice per group. (I) Bioluminescence flux of tumor cell growth following different treatments on the indicated days after CAR‐T cell infusion. Dots represent individual mice; bars represent mean ± s.d. Statistical significance among groups was determined using one‐way ANOVA, n = 5 mice per group. (J) Representative H&E staining images of mouse liver isolated from mice bearing lymphoma in each group. n = 5 mice per group. Scale bar, 100 µm. (K) Schematic of the experimental set‐up for systemic injection of stCAR and auxCAR T cells. First, 2 × 10^5^ Nalm6 cells labeled with the lipophilic near‐infrared fluorescent dye DiR (1,1'‐dioctadecyl‐3,3,3',3'‐tetramethylindotricarbocyanine iodide) in vitro were intraperitoneally (i.p.) injected into C‐NKG mice. After 4 days, 2 × 10^6^ UTD (Untransduced), stCAR T, or auxCAR T cells were injected intraperitoneally (i.p.) Mice received cvxIAA by oral gavage q.o.d (every other day) for the duration of the study. The auxin‐induced auxCAR T‐cell group was designated as auxCAR+, and the group not subjected to cvxIAA induction was designated as auxCAR‐. (L) Statistical analysis of mouse body weights of mice measured on day 35. Dots represent individual mice; bars represent mean ± s.d. One‐way ANOVA analysis was used for statistical significance, n = 5 mice per group. (M) CD19^+^ marking of tumor cells in ascites. (N) DiR fluorescent imaging of tumor cell growth following different treatments on the indicated days after CAR‐T cell infusion. (O) Survival of indicated treatment mice.

Our in vivo mouse model showed that, after cvxIAA induction via oral gavage every other day, anti‐CD19 auxCAR‐T cells effectively controlled systemic B‐cell lymphoma. This was evident from the significant reduction in the proportion of CD19‐positive Raji tumor cells in the bone marrow (Figure 5C and Figure S7A). In the absence of cvxIAA, auxCAR‐T cells showed no effect on the inhibition of tumor growth, a result comparable to the control group, consisting of peripheral T cells without CAR transduction (UTD: Untransduced group). At day 21, mice in the UTD and auxCAR‐ groups lost more than 20% of their body weight, whereas the stCAR and auxCAR+ groups maintained their body weight, indicating reduced cancer‐induced cachexia (Figure 5H). Notably, Longitudinal bioluminescence imaging revealed that the in vivo anti‐tumor activity of cvxIAA‐induced auxCAR T cells was slightly improved but not significant to that of stCAR T cells, with both substantially reducing tumor burden compared with UTD and auxCAR‐ controls (Figure 5I and Figure S7F).

Importantly, both auxCAR‐T cells with auxin induction and CD19‐CAR‐T cells, detected in the bone marrow (Figure 5D and Figure S7B), peripheral blood (Figure 5E and Figure S7C), and spleen (Figure 5F and Figure S7D) of mice, underwent efficient expansion and reconstitution, with similar expansion frequencies in both groups. Notably, the frequency of auxCAR T cell expansion was markedly elevated in the auxin‐induced group compared to the non‐induced group, indicating that auxCAR undergoes substantial activation and proliferation in vivo following cvxIAA induction, thereby reaffirming the robust efficiency of the cvxIAA induction system. Consistent with in vitro observations, auxCAR‐T cells exhibited a higher frequency of central memory T cells (TCM) than CD19‐CAR‐T cells following antigen stimulation (Figure 5G and Figure S7E). This preservation of TCM predominance suggests auxCAR‐T cells maintain a therapeutically favorable memory phenotype in vivo, which correlates with slightly improved but not significant antitumor responses in vivo (Figure 5I). Histological staining also revealed that tumor cell infiltration in the liver was markedly reduced in the auxCAR+ group, with efficacy comparable to that of stCAR‐T cells (Figure 5J).

We also used an additional animal model, the Nalm6 intraperitoneal tumor burden model (Figure 5K). Nalm6 cells were labeled with DiR dye and inoculated intraperitoneally. After administration of cvxIAA (50 mg/kg) by oral gavage every other day, mice in the UTD and auxCAR‐ groups lost more than 20% of body weight by day 21, whereas the stCAR and auxCAR+ groups maintained their body weight, indicating effective tumor control (Figure 5L). Longitudinal DiR fluorescence imaging showed that stCAR and auxCAR+ T cells efficiently controlled Nalm6 tumors on days 21, 28, and 35 post‐tumor inoculation, compared with the UTD and auxCAR‐ groups. Although the DiR signal was sharply downregulated in all groups by day 7 compared with day 35, likely due to dilution from cell proliferation, the signal in the auxCAR‐ and stCAR groups remained considerably weaker than in the UTD and auxCAR+ groups at day 35, indicating that the weaker signal resulted from tumor cell killing rather than dilution (Figure 5N). In addition, the stCAR and auxCAR+ groups showed a significant reduction in CD19‐positive tumor cells in ascites compared to the UTD and auxCAR‐ groups, with the auxCAR+ group exhibiting an even greater reduction than the stCAR group, suggesting more durable anti‐tumor efficacy (Figure 5M). Survival analysis revealed that all mice in the UTD and auxCAR‐ groups died between days 35 and 40, whereas mice in the auxCAR+ and stCAR groups survived until approximately day 50. The auxCAR+ group showed a trend toward prolonged survival compared with the stCAR group (Figure 5O). Consistent with the in vitro anti‐exhaustion data, these survival curves demonstrate that auxCAR+ T cells have improved anti‐exhaustion and tumor‐killing capacity in vivo relative to stCAR T cells.

Finally, to evaluate whether auxin could mitigate CRS induced by auxCAR T cells, we established an in vitro assay (Figure 6A) in which CAR T cells were cocultured with monocytes and target tumor cells in the presence of DMSO (vehicle) or auxin, and IL‐6 levels were measured in the supernatant. On day 12, T cells and monocytes were seeded together with A431‐CD19 tumor cells. This procedure was repeated once with fresh A431‐CD19 cells on days 13 and 14. Supernatants were collected on days 14 and 15. In serial cocultures, while auxin, but not DMSO, triggered IL‐6 production, administration of auxin led to a marked reduction in IL‐6 secretion in the auxCAR T group at 48 and 72 h after co‐culture, relative to the stCAR T group (Figure 6B, middle). Conversely, neither compound influenced IL‐6 production in the stCAR T co‐culture group (Figure 6B, right). These in vitro data suggest that auxin administration could mitigate CRS triggered by auxCAR T cells in vivo.

**FIGURE 6:**
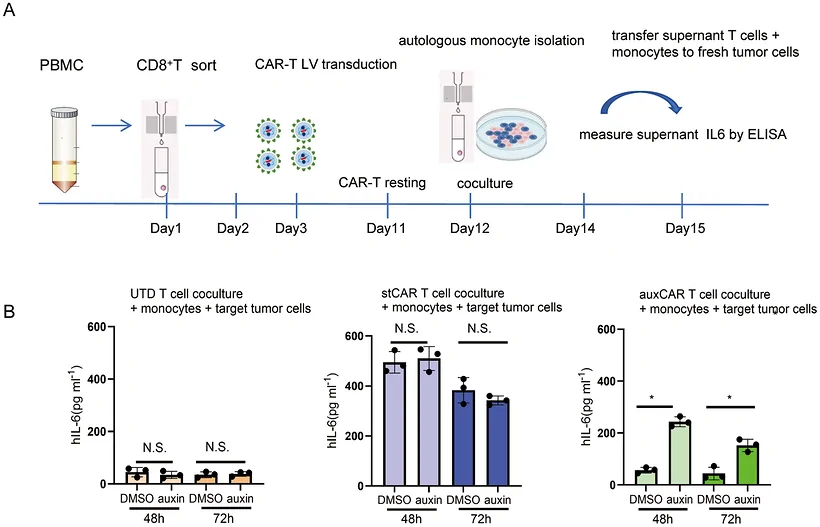
CRS potential of auxCAR vs. stCAR T cells. (A) Schematic of the CRS assay; (B) CRS potential assay by quantification of IL‐6 production upon co‐culture of UTD (left), stCAR (middle), auxCAR (right) on day 14 and 15, and co‐culture with autologous monocytes and cognate tumor cells. Autologous monocytes were isolated (and donor T cell matched) on the assay day. Cocultures were established in the presence of 1 µM auxin (n = 3 donors). A one‐way ANOVA was used to determine statistical significance by comparing all groups within each day.

In summary, the in vitro functions of anti‐CD19 auxCAR‐T cells were dependent on the presence of auxin and the specific binding of the target antigen. This resulted in precise, targeted cell killing while minimizing the risk of inflammatory cytokine storm side effects.

## 3 Discussion

In this study, we introduce a synthetic biology approach that leverages plant hormone signaling components to engineer a novel inducible on‐switch in CAR‐T cell immunotherapy. This auxCAR system achieves efficient, robust, and reversible T cell activation in response to auxin. Its tunable activation mechanism enables precise, on‐demand control over T cell responses, providing a modular platform for dynamic immune regulation. This controllability reduces the risks associated with prolonged stimulation, such as cytokine storms and off‐target toxicity, and offers a strategic advantage in managing adverse effects. The synthetic design of the auxCAR system appears to enhance resistance to T cell exhaustion, likely by maintaining a balanced activation state, while the increased proportion of central memory T cells (TCM) suggests an engineered capacity for sustained, long‐term anti‐tumor activity. In vivo validation in a hematolymphoma model demonstrated that auxCAR‐T cells can specifically and effectively target CD19‐positive tumors, underscoring the therapeutic potential of integrating synthetic biology tools for precise immune modulation. Unlike conventional CAR‐T systems that rely on persistent activation or prior switchable CAR‐T with immunogenicity, this auxCAR‐T system provides a safe and adaptable method for controlling T cell responses, paving the way for next‐generation, programmable immunotherapies with enhanced safety and efficacy.

Compared with existing switchable CAR‐T systems, several distinctive features position auxin‐inducible switchable CAR‐T (auxCAR‐T) cells as an improved alternative to current switchable CAR‐T systems. First, the auxin‐inducible degron (AID) system enables ultrafast, reversible control of CAR‐T activity [53], on the order of minutes, far surpassing the slower kinetics of antibody‐based switches (e.g., FITC‐ or biotin‐tagged systems) [18] or chemical dimerizers (e.g., rapamycin analogs like rimiducid) [16]. This rapid on/off switching is critical for mitigating acute toxicities such as cytokine release syndrome (CRS) or neurotoxicity (ICANS) [61], which remain dose‐limiting challenges in conventional and even some switchable CAR‐T therapies [62]. Second, the plant‐derived auxin (indole‐3‐acetic acid, IAA) is non‐immunogenic in humans [41], eliminating a major drawback of antibody‐ or protein‐based switches that often trigger neutralizing anti‐drug antibodies (ADAs) [63]. For instance, AMG 119's FITC‐based switch has shown immunogenicity in early trials [64], while auxCAR‐T avoids this pitfall entirely by leveraging a small molecule endogenous to human metabolism. Third, auxin can be administered orally or intravenously, offering unparalleled flexibility compared to light‐controlled CAR‐T systems (e.g., OptoCARs), which require invasive implanted fiber optics [21] or antibody switches that necessitate intravenous infusion [19]. In addition, the AID system's modularity allows seamless integration with advanced synthetic biology tools, such as logic‐gated circuits or hybrid degradation systems [40, 53]. For example, auxin can be combined with PROTACs to simultaneously degrade inhibitory checkpoint proteins (e.g., PD‐1) or paired with suicide genes (e.g., iCasp9) for multi‐layered safety [17, 65]. This adaptability contrasts with rigid, single‐mechanism platforms like UniCAR (peptide‐tagged switches) [66] or SUPRA CAR (leucine zipper adapters) [67], which lack such combinatorial potential. Furthermore, auxin's natural metabolic clearance reduces off‐target toxicity risks, unlike rapamycin analogs, which may inhibit mTOR signaling [15], or synthetic dimerizers with poorly characterized long‐term effects. Therefore, auxin‐inducible CAR‐T cells represent a promising next‐generation switchable platform, addressing several cardinal limitations of current clinical candidates: slow kinetics, immunogenicity, and impractical delivery requirements.

While auxin‐based sCAR‐T presents a groundbreaking alternative, challenges such as low‐level leaky degradation and the need for optimized auxin dosing remain. Future studies will focus on further refining auxin‐inducible sCAR‐T systems to enhance their precision, safety, and therapeutic efficacy. Future study includes further optimizing auxin‐inducible sCAR‐T systems to enhance their precision, safety, and efficacy. This involves refining the pharmacokinetics of auxin analogs [68], integrating additional safety features such as a dual‐control mechanism [69], and developing multi‐layered regulation strategies to better control T‐cell activity. Exploring combination therapies with checkpoint inhibitors or cytokine modulators could address tumor immunosuppressive microenvironments [70] and improve therapeutic outcomes. Additionally, efforts should focus on addressing tumor heterogeneity and ensuring long‐term stability and persistence of engineered cells. Advancing scalable manufacturing processes and establishing clear regulatory pathways will be essential for translating these innovations into clinical settings, ultimately enabling personalized, controllable, and safer cancer immunotherapies.

Distinct from the safety of the inducer itself, the AFB1 and IAA7 protein domains incorporated into the auxCAR construct are derived from plant proteins and may therefore present immunogenicity risks in human recipients. These non‐human domains could potentially elicit anti‐drug antibody responses, which might neutralize therapeutic T cell function or provoke immune‐mediated adverse events. Accordingly, while the current study establishes proof of concept using the AFB1‐IAA7 degron system, clinical translation will likely require humanization of these domains or alternative protein engineering strategies to minimize immunogenicity risk. Ongoing and future efforts will focus on identifying or engineering minimally immunogenic degron pairs that retain the rapid, reversible control demonstrated here.

Another limitation of this study is the use of an intraperitoneal tumor burden model, where both Nalm6 cells and CAR‐T cells were injected intraperitoneally. This approach models localized peritoneal disease rather than the systemic dissemination typical of leukemia after intravenous injection. As such, the ability of CAR‐T cells to control leukemia in this context may not fully represent their efficacy against systemic disease, a point that should be considered when interpreting these findings.

In conclusion, with its unique combination of rapid responsiveness, enhanced safety, and versatile control, auxCAR‐T therapy is well‐positioned to outperform existing switchable CAR‐T systems in both clinical effectiveness and resistance to T cell exhaustion, particularly in tumors where precise regulation is critical [70]. As the field advances toward allogeneic, off‐the‐shelf CAR‐T products, the auxin system's compatibility with universal donor cells further reinforces its potential as a transformative technology in controllable immunotherapy [71]. This innovation paves the way for safer, more adaptable, and highly programmable CAR‐T therapies, ultimately broadening the horizons of personalized cancer treatment.

## 4 Methods

Methods, including statements of data availability and associated analysis codes and references, are available in the online methods.

### 4.1 Online Methods

#### 4.1.1 Material and Methods

##### 4.1.1.1 Molecular Cloning and Plasmid Construction

To construct plasmids encoding IAA7 R chain, gBlocks gene fragments were synthesized by SANGON Technologies to generate the following complementary DNA sequences: CD8 leading signal peptide (sp), anti‐CD19 scFv or anti‐HER2 scFv, CD8α transmembrane domain, CD8α hinge region, and the costimulatory receptor 4‐1BB. The synthetic IAA7 block was first amplified by standard PCR with ApexHF HS DNA Polymerase FS Master Mix (dye plus) (AG12206, Accurate Biotechnology (Hunan)Co., Ltd., China), TransTaq DNA Polymerase High Fidelity (AP131‐01, Transgene), and then inserted downstream of 4‐1BB. For live cell imaging constructs, we generated fusion protein versions with Venus.

To construct plasmids encoding AFB1 S chain, we sequentially introduced NES (nuclear export signal), DAP10, CD8α transmembrane domain, CD8α hinge region, and the co‐stimulatory receptor 4‐1BB4‐1BB, human CD3ζ intracellular chain derived from gBlocks gene fragments and inserted AFB1 upstream of CD3ζ. To facilitate further CAR enrichment, component I was further modified by inserting a T2A Thy1.1 for cell sorting and enrichment. For live cell imaging constructs, we generated fusion protein versions with mScarlet.

##### 4.1.1.2 Cell culture, Transfection and Confocal Imaging

An NFAT‐GFP reporter Jurkat cell line (Clone E6‐1, ATCC, TIB‐152) was used to examine T cell activation by GFP expression as previously described. The human cancer cell lines 293T (ATCC, CRL‐3216), K562 (ATCC, CCL‐243), Raji (ATCC, CCL‐86), Nalm6 (ATCC, CRL‐3273) were obtained from the American Type Culture Collection (ATCC) and maintained in RPMI 1640 medium supplemented with 1 mM glutamine, 10% fetal bovine serum (FBS), 100 U mL^−1^ penicillin/streptomycin (Gibco). HeLa (ATCC, CRM‐CCL‐2), SK‐OV‐3 (ATCC, HTB‐77), HEK293T cells (ATCC, CRL‐3216), and A431 (ATCC, CRL‐1555) from ATCC were maintained in DMEM supplemented with 10% FBS and 1% P/S. For confocal imaging, the auxCAR system, comprising IAA7‐Venus (R chain) and AFB1‐mScarlet (S chain) fusion constructs, was introduced into HeLa cells via Lipofectamine 2000‐mediated transfection following standard procedure. Confocal microscopy was performed 36 h post‐transfection using a Nikon A1 inverted confocal system equipped with 488 and 561 nm laser lines. The acquired images were processed and analyzed with Nikon NIS‐Elements (version 5.22) and ImageJ analysis packages

##### 4.1.1.3 Lentiviral Production

The second‐generation lentiviral packaging plasmid PMD2.G, PSPAX2, and the plasmid of interest were co‐transfected at a ratio of 1:2:3 into HEK 293T cells using either the Lipofectamine 2000 Transfection Reagent following the manufacturer's protocol. The supernatant containing the lentiviral particles 36, 48, and 72 h post‐transfection was collected together and filtered through 0.45 uM Polyvinylidene Fluoride (PVDF) filters. The supernatant was centrifuged at 4°C for 2 h at 25 000 rpm, resuspended with IMDM medium, and aliquots can be stored at −80°C. Viral titers (transduction units) were calculated based on cell surface expression of Thy1.1 measured by flow cytometry 72 h post‐transduction

##### 4.1.1.4 Isolation and Culture of Primary Human T Cells

PBMCs were isolated from blood samples of healthy blood donors by density gradient centrifugation using Ficoll‐Paque Plus medium (Cat# GE17‐1440‐02, MERCK). The PBMCs were washed once with PBS and resuspended at a cell density of 1 × 10^6^ cells/mL in PBS supplemented with 2% FBS and 1 mM EDTA. CD3^+^ T cells from the PBMCs were enriched by magnetic cell sorting using pan T positive selection kits (BioLegend). The purity of the CD3^+^ cells was measured using flow cytometry by staining with FITC‐conjugated anti‐human CD3 (Cat# 300405, BioLegend). The purified pan T cells were cultured in T cell medium‐RPMI l640‐glutamine containing medium supplemented with 10% FBS, 1 mM sodium pyruvate (Gibco), 10 mM HEPES (Gibco), 0.55 µM 2‐mercaptoethanol (Gibco), 1% P/S. Recombinant human IL‐2 (Cat# RP01039, ABclonal) was added at a final concentration of 100 Unit/mL^−1^ to expand T cells.

##### 4.1.1.5 Generation of CAR‐T Cells

The purified T cells were activated with ActiveMax Human T cell Activation/Expansion CD3/CD28 beads, premium grade (Cat# MBS‐C001‐10 mg, Acrobiosystems, 1:1 bead‐to‐cell ratio) in T cell medium. 36–48 h post‐activation, the condensed stCAR/auxCAR lentivirus was added to each well precoated with retroNectin (T100A, Takara Bio) and spinoculated at 2000 rpm at 32°C for 1 h. The medium was changed 12 h post‐transduction. The transduction efficiency was evaluated by flow cytometry 48–72 h post transduction.

##### 4.1.1.6 Flow Cytometry

All cells in the culture were washed once with FACS buffer before staining with antibodies. After a 20 min incubation at 4°C in the dark, the cells were washed twice with FACS buffer and analyzed with a CytoFLEX S instrument. stCAR and auxCAR expression was measured by staining with an Alexa Fluor 647 Conjugate anti‐mouse IgG (H+L), F(ab')2 Fragment antibody (Cat# 4410, Cell Signaling Technology) and PE anti‐rat CD90/mouse CD90.1 (Thy1.1) antibody (Cat# 202523, BioLegend). Jurkat T cells transduced with stCAR/auxCAR were co‐cultured with either CD19^−^ (K562) or CD19^+^ (Raji) tumor cells at a ratio of 1:2 in 96‐well flat‐bottom microplates (Corning). Auxins were added at a gradient concentration as indicated in the figures. 12 h post co‐culture, the cells were washed once and stained with an APC‐conjugated anti‐human CD69 antibody (Cat# 985206, BioLegend) at 4°C in the dark for 20 min in FACS buffer. The fluorochrome‐conjugated antibodies included anti‐CD3 (E‐AB‐F1001C, Elabscience), anti‐CD8 (A28209PM, ABclonal), anti‐CD45RA (A22571, ABclonal), anti‐CCR7 (A27683, ABclonal),, anti‐LAG‐3 (A28468, ABclonal), anti‐PD‐1 (A25730, ABclonal),. The data were analyzed with FlowJo 10 software.

##### 4.1.1.7 3D Complex Structure Prediction

We used the current mainstream 3D complex prediction tools (AlphaFold3, Proteinix, Chai‐1, Boltz‐1) to make structural predictions for the 3‐membered complex AFBs proteins (AFB1, AFB2, AFB5, TIR1), IAAs proteins (IAA3, IAA5, IAA7, IAA8), and small molecules of IAA. Each combination achieves 5 predicted structures. Details can be found in Supporting Information and Methods.

##### 4.1.1.8 Fluorescence Resonance Energy Transfer (FRET) Analysis of Dimerization

For flow cytometry‐based FRET analysis of IAA7 R chain and AFB1 S chain dimerization, the auxCAR‐transduced Jurkat cells were stained with a PE‐conjugated MYC tag Monoclonal antibody (Cat# PE‐60003‐2, Proteintech) and the Alexa Fluor 647 Conjugated anti‐mouse IgG (H+L), F(ab')2 Fragment antibody (Cat # 4410, Cell Signaling Technology). The cells were treated with or without auxin, and the FRET signal was detected in the PE‐Cy5 channel within the gated scFv/myc double‐positive cells. Cell signal was recorded using the BD Beckman CytoFLEX S and analyzed with FlowJo 10 software.

##### 4.1.1.9 Detection of IAA7/AFB1 Dimerization by Fluorescence Lifetime Imaging Analysis

FRET‐FLIM analysis was conducted on a Nikon A1 confocal system equipped with a PicoQuant FLIM module. Transiently transfected cells were imaged through 488/561 nm filters, followed by fluorescence lifetime measurements using a SPAD detector. System calibration involved determining the instrument response function (IRF) with Convallaria specimens before and after each session per manufacturer guidelines. Fluorescence decay kinetics were analyzed in SymPhoTime 64 via bi‐exponential reconvolution fitting to the IRF, with mean lifetimes (τ) displayed in FLIM images. We quantified Venus‐tagged IAA7 and Scarlet‐labeled AFB1 lifetimes in co‐transfected cells with or without 3‐Indoleacetic acid (IAA) (Cat# I2886, MERCK) treatment, acquiring ≥50 measurements per condition across three technical replicates.

##### 4.1.1.10 In Vitro Quantification of NFAT‐GFP Activity of Jurkat‐GFP T Cells

Jurkat T cells expressing stCARs, auxCAR (2 × 10^5^ cells per well) were co‐cultured with cognate CD19^+^ Raji cells, A431‐CD19 cells, or non‐cognate CD19^−^ K562 cells at the indicated effector T cell/target cell (E/T) ratio of 3:1 in 48‐well plates. Cells were harvested after 16 h of co‐culture, and GFP signal was measured by flow cytometry. Data plots were generated using FlowJo 10 software and the Prism software (version10, GraphPad).

##### 4.1.1.11 Cytotoxicity Assay and Cytokine Release Assay

Human T cells engineered to express either stCAR or auxCAR were co‐cultured with cognate CD19^+^ Raji or SK‐OV‐3 tumor cells, with both populations exhibiting >90% viability. Target cells were co‐cultured with effector T cells at various 3:1, 1:1, or 1:3 effector‐to‐target (E/T) ratios in T cell medium using 48‐well plates. CAR‐T cells and target cells were co‐cultured for 24 h at 37°C, 5% CO2. Cells were harvested and labeled with 100 nM SYTOX Blue dye (Cat# S34857, Invitrogen) in FACS buffer for 20 min at 4°C. Following two washes, cells were resuspended in FACS buffer and analyzed on BD Beckman CytoFLEX S. Data plots were generated using FlowJo 10 software and the Prism software (version10, GraphPad).

For the measurement of cytotoxicity, briefly, we first gated on FSC‐A and SSC‐A. Singlets were gated using FSC‐H vs. FSC‐A. T cells were then strictly excluded by gating on the CD3^−^ population. Dead cells within this gated target cell population were identified by Sytox Blue staining, and the percentage of Sytox Blue^+^ cells was calculated.

For the cytokine secretion assay, the supernatants were collected 24 h post co‐culture, and IFN‐γ and IL‐2 levels were measured with a human IL‐2 precoated enzyme‐linked immunosorbent assay (ELISA) kit (cat# 1110202, DAKAWE) and a human IFN‐γ precoated ELISA kit (cat# 1110002, DAKAWE) following the manufacturer's instructions.

##### 4.1.1.12 Repeated Tumor Antigen Stimulation Assay

stCAR or auxCAR T cells (0.25 million) were co‐cultured with A413‐CD19 cells (0.25 million) at a 1:1 effector‐to‐target (E:T) ratio in a mixture of half T cell medium and half A431‐CD19 culture medium with approximately 100 U/mL recombinant human IL‐2. Auxin (1 uM) was added to the supernatant every 48 h. Every 48 h, the supernatant containing CAR‐T cells was collected from the co‐culture plate and centrifuged. Fresh A431‐CD19 cells (0.25 million) were added to the T cells daily to maintain repeated stimulation. Cells were harvested on day 10 of the assay. Cytotoxicity and T cell exhaustion markers were measured by flow cytometry using the following fluorochrome‐conjugated antibodies: FITC anti‐rat CD90/mouse CD90.1 (Thy1.1) antibody (Cat# 11‐0900‐81, eBioscience), anti‐TIM‐3 (Cat# 345012, BioLegend)., anti‐PD‐1(Cat# 379209, BioLegend). For the gating strategy for PD1^+^/ TIM3^+^, briefly, lymphocytes were gated on FSC‐A/SSC‐A. Singlets were then identified using FSC‐H/FSC‐A, followed by selection of live cells (DAPI^−^). CAR^+^ (Thy1.1^+^) cells were then identified from the live single‐cell population. Percentages shown represent the frequency of PD‐1^+^ or TIM‐3^+^ cells within the live CAR^+^ T cell population.

For the measurement of cytotoxicity, briefly, we first gated on FSC‐A and SSC‐A. Singlets were gated using FSC‐H vs. FSC‐A. T cells were then strictly excluded by gating on the CD3‐ population. Dead cells within this gated target cell population were identified by Sytox Blue staining, and the percentage of Sytox Blue^+^ cells was calculated. The data were analyzed with FlowJo 10 software.

##### 4.1.1.13 RNA‐seq Data Processing and Analysis

Raw sequencing reads were quality‐checked and preprocessed using Fastp (a high‐efficiency tool that performs integrated quality control, adapter trimming, filtering, deduplication, and base correction in a single‐pass workflow). The processed reads were then aligned to the GRCh38.p14 reference genome (Homo sapiens) using STAR v2.7.11b, followed by gene‐level read quantification with featureCounts (from the Rsubread v2.16.1 R package). Downstream analysis included normalization, differential expression testing, and principal component analysis (PCA) using DESeq2 v1.42.1. Visualization was performed with pheatmap v1.0.12 (heatmaps) and EnhancedVolcano v1.20.0 (volcano plots). All statistical computations and graphics were generated in R v4.3.1.

##### 4.1.1.14 Animal Experiments

For the Raji lymphoma mouse model, 6–8‐week‐old female C‐NKG mice (NOD.Cg‐Prkdc^scid^Il2rg^em1cya/^Cya, C001316, Cyagen Biosciences) were tail vein injected (i.v.) with 2 × 10^5^ Raji cells per mouse. After 4 days, 2 × 10^6^ stCAR or auxCAR T cells per mouse were systemically administered by tail vein injection (i.v.) in one dose. For the Nalm6 intraperitoneal tumor burden model, 2 × 10^5^ Nalm6 cells per mouse were labeled with the lipophilic near‐infrared fluorescent dye DiR (1,1'‐dioctadecyl‐3,3,3',3'‐tetramethylindotricarbocyanine iodide) in vitro and washed three times, then intraperitoneally (i.p.) injected into the peritoneal cavity of 6–8 week‐old female C‐NKG mice. After 4 days, stCAR T or auxCAR T cells per mouse were injected intraperitoneally (i.p.). Bioluminescent imaging or DiR fluorescent imaging was performed weekly using the IVIS Imaging System (PerkinElmer) with accompanying analysis software Prior to CAR‐T cell administration, mice were randomized based on quantitative tumor radiance measurements. Treatment with 5‐(3‐methoxyphenyl)indole‐3‐acetic acid (cvxIAA) (Y56681, Shanghai Yuanye Bio‐Technology Co., Ltd) (50 mg kg^−1^) was administered by oral gavage every other day [59]. All animals were monitored daily for general health status, including body weight, mobility, and grooming. Body weight was recorded every 7 days throughout the study period, and percentage weight change relative to the initial weight was calculated for each animal. No animal in any experimental group exhibited a body weight loss exceeding 15% of its initial weight at any time point. The full time‐course body weight data are provided in Figure S7E.

Blood samples were collected from the retro‐orbital plexus of all treated mice using 1.5 mL tubes pre‐coated with 1 mM EDTA, after which red blood cell lysis was performed. The cells were stained with a PE anti‐rat CD90/mouse CD90.1(Thy1.1) Antibody (Cat#202523, BioLegend) to leverage CAR expression. On day 21, mice were euthanized for organ isolation and phenotypic analyses. Single‐cell suspensions from bone marrow and spleen were prepared for flow cytometric analysis.

##### 4.1.1.15 Histological Assay

Mice were sacrificed at the indicated time point, and the tissue samples were sectioned, fixed using 10% formalin, dehydrated through ethanol treatment, and subsequently paraffin‐embedded. Serial 4 µm sections were obtained from the tissue samples using a microtome. The sections were subjected to hematoxylin‐eosin (HE) staining. Subsequently, the samples were examined, and images were captured using an Olympus BX53 optical microscope. Image analysis was performed with Saiviewer software.

##### 4.1.1.16 Statistical Analyses

Quantitative data are expressed as mean ± s.d. Statistical analysis was performed by unpaired Student *t*‐test for comparisons between 2 groups and by one‐way ANOVA, followed by Bonferroni test for comparisons between >2 groups. For analysis of mouse survival curve and signs of morbidity, an extension of the Fisher test (Freeman–Halton) was used via a 3 × 3 contingency table. A value of *p* < 0.05 was considered statistically significant. Significance levels were denoted as follows: ^*^ *p* < 0.05, ^**^ *p* < 0.01, and ^***^ *p* < 0.001. All statistical analyses and graphical representations were generated using GraphPad Prism software (version 10.0).

## Author Contributions

**Rongkun Tao**: Writing – review and editing, Writing – original draft, supervision, formal analysis. **Sirui Zheng**: formal analysis, project administration, software, methodology. **Yanke Zhong**: investigation, project administration. **Yongxia Niu**: investigation, project administration. **Min Zhang**: project administration, investigation. **Chen Yang**: investigation, project administration. **Xuan Wei**: writing – review and editing. **Jing Yang**: software. **Fuyu Duan**: writing – review and editing, visualization. **Zhenbiao Yang**: conceptualization. **Qizhou Lian**: conceptualization, writing – original draft, writing – review and editing. **Peng Zhang**: investigation, project administration. **Hongxiang Zeng**: writing – review and editing, writing – original draft, funding acquisition, formal analysis, project administration, conceptualization, investigation, software. **Lei Zheng**: data curation, software, project administration. **Zhaoxi Sun**: visualization, software, project administration. **Xiang Zhou**: investigation, methodology. **Xiang Ma**: investigation.

## Funding

This work was in part supported by National Key Research and Development Program of China (2023YFA0914904) to QL and HZ, Shenzhen‐Hong Kong joint Science and Technology Innovation Program (SGDX20240115113100001) to QL and HZ, and National Natural Science Foundation of China (NSFC, Grant No. 82303094) to HZ, National Natural Science Foundation of China Grant (W2431026) to ZY, Shenzhen Science and Technology Program (Grant No. SYSRD20250529113008011) to HZ.

[Correction added on 9 September 2026 after first online publication: The funding grant number “3241101698” was incorrect and has been corrected to “W2431026”.]

## Ethics Approval

Human peripheral blood samples were obtained from healthy donors under a protocol approved by the Scientific Research Ethics Committee of Guangzhou Women and Children's Medical Center, and written informed consent was obtained from all participants. All animal procedures were performed in accordance with the guidelines approved by the Institutional Animal Care and Use Committee of Animal Facility (IACUC) of the Brain Cognition and Brain Disease Institute (BCBDI), Shenzhen Institutes of Advanced Technology (SIAT), Chinese Academy of Sciences (CAS) (IACUC Approval Number: No. SIAT‐BSI‐IRB‐250114‐HCS‐ZHX‐A0095‐01).

## Conflicts of Interest

The authors declare no competing financial interests.

## Supporting information




**Supporting File**: advs77020‐sup‐0001‐SuppMat.docx.

## Data Availability

The RNA‐seq data generated in this study are deposited in the NCBI Gene Expression Omnibus under accession number (GSE301774) [NCBI tracking system #25255668]. The raw sequencing data supporting the findings of this study are available from the corresponding authors upon reasonable request.
