# Supplementary material for: Repurposing a Small Molecule Plant Hormone as a Tunable ON‐Switch for CAR‐T Cell Immunotherapy
Source: Adv Sci (Weinh). 2026 Aug 11;13(61):e77020. doi: 10.1002/advs.77020 (PMC13460283; doi:10.1002/advs.77020)
Supplement: Supplementary file 1 [file ADVS-13-e77020-s001.docx]

**Supplemental information**

**Supplementary Fig. 1 The predicted structures and molecular simulation dynamics results of AFBs-IAAs.**

A) The predicted 3D structure of 16 combinations by AlphaFold3. B) Stabilizing effects contributed by protein residues under IAA-bound and unbound conditions.

**Supplementary Fig. 2 Orthogonal Activation System for Auxin and Its Derivatives**

A, B, C) Flow cytometric representative data revealed auxin (1μM) (A) and 5-Ph-IAA (1μM) (B) and cvxIAA (1μM) (C) induced GFP reporter expression in HEK293T cells co-expressing AFB1 WT/ AFB1 F75G-BD and IAA7 WT/IAA7 P87S -AD.

**Supplementary Fig. 3 Immune Gene Expression Profiles Are Similar Between auxCAR and stCAR.**

A) Principal Component Analysis (PCA) in stCAR and auxCAR tranduced primary CD8^+^ T cells. B) Box line diagram and volcano map of differentially expressed genes in stCAR and auxCAR transduced primary CD8^+^ T cells. C) Flow cytometric representative histograms showing CD69 expression in Jurkat cells treated with 1 μM auxin or 1μM cvxIAA. D) Cell proliferation counts in Jurkat cells treated with 1 μM auxin or 1μM cvxIAA.

**Supplementary Fig. 4 Assessment of auxCAR-T cell performance in Jurkat T cells.**

A-B) Flow cytometric representative data of NFAT-GFP reporter activity in Jurkat T cells expressing stCAR and auxCAR co-cultured with tumor cells with hCD19^+^ Raji cells (A) or A431-CD19^+^ (B) with the indicated auxin concentration.

C) Flow cytometric representative data of NFAT-GFP reporter activity in AuxCAR expressing Jurkat T cells inactivated post auxin removal and reactivated upon the reintroduction of auxin and subsequent co-culture with CD19^+^ target tumor cells.

D) Flow cytometric representative data of NFAT-GFP reporter activity in Jurkat T cells expressing stCAR HER2 or auxCAR HER2 co-cultured with SK-OV-3 tumor cells in the presence of indicated auxin concentration.

E) Flow cytometric representative data of NFAT-GFP reporter activity at the indicated time points following auxin removal in Jurkat T cells expressing auxCAR and co-cultured with SK-OV-3 tumor cells. The auxCAR T cells were induced with 1 μM auxin.

**Supplementary Fig.5 Comparison of the *in vitro* performance of AuxCAR-transduced primary T cells against hematological and solid tumors.**

A) Gating strategy of cytotoxicity of T cells against target cells.

B-C) Flow cytometric representative data of SYTOX blue positive cells to determine cytotoxicity auxCAR-19 T cells cocultured with Raji cells (B) and auxCAR-HER2 T cells co-cultured with SK-OV-3 cells (C) with gradient concentration of auxin indicated. stCAR-transduced T cells were used as a positive control in all experiments.

D-E) Cytotoxicity against target cells was evaluated at the indicated effector-to-tumor (E:T) ratios (3:1, 1:1, 1:3) using auxCAR19 T cells from healthy donor 2 (D) and donor 3 (E) activated with 1μM auxin, stCAR-transduced T cells were used as a positive control in all experiments. n=3 independent experiments (with 1 donor per experiment).

**Supplementary Fig. 6 Comparison of auxCAR T cell exhaustion and killing with stCAR T cells**

A) Gating strategy of PD-1^+^/TIM-3^+^ cells in repeated tumor stimulation co-culture assays.

B) Gating strategy of cytotoxicity of T cells against target cells in repeated tumor stimulation co-culture assays.

C, D, E, F, Flow cytometric representative data of TIM-3 (C, E), PD-1 (D, F) on CAR T cells before (C, D) and after (E, F) exposure to the repeated tumor stimulation assay.

G, H, Statistical Analysis of PD-1^+^TIM-3^+^ auxCAR19 T cells from 3 healthy donors before (G) and after (H) repeated tumor stimulation co-culture assays. stCAR19 T cells were used as a positive control in all experiments. n=3 independent experiments (with 1 donor per experiment).

I, J, Statistical Analysis of killing efficiency of auxCAR19 T cells from 3 healthy donors before (I) and after (J) repeated tumor stimulation co-culture assays. stCAR19 T cells were used as a positive control in all experiments. n=3 independent experiments (with 1 donor per experiment).

**Supplementary Fig. 7 AuxCAR-positive primary CD8^+^ T cells delayed Raji B-cell lymphoma and Nalm6-B cell leukemia burden *in vivo.***

 A, B, C, D, E,) Flow cytometric representative data of of CD19^+^ Raji tumor cells in the bone marrow (A) were determined on day 21 post injection and CAR^+^ T cell persistence in the bone marrow (B), blood (C), spleen (D), central memory (TCM: CD45RA^-^CCR7^+^) cells (E) gated from CAR^+^ T cells was determined on day 14 post injection. n = 5 mice per group.

(F) Bioluminescence imaging of whole mice in each group, indicating tumor burden at representative time points..

**Supplementary Tables**

**Supplementary Tables 1: Amino acid sequence of IAA7**

MFSETVDLMLNLQSNKEGSVDLKNVSAVPKEKTTLKDPSKPPAKAQVVGWPPVRNYRKNMMTQQKTSS

**Supplementary Tables 2: Amino acid sequence of AFB1**

MGLRFPPKVLEHILSFIDSNEDRNSVSLVCKSWFETERKTRKRVFVGNCYAVSPAAVTRRFPEMRSLTLKGKPHFADYNLVPDGWGGYAWPWIEAMAAKSSSLEEIRMKRMVVTDECLEKIAASFKDFKVLVLTSCEGFSTDGIAAIAATCRNLRVLELRECIVEDLGGDWLSYFPESSTSLVSLDFSCLDSEVKISDLERLVSRSPNLKSLKLNPAVTLDGLVSLLRCAPQLTELGTGSFAAQLKPEAFSKLSEAFSNCKQLQSLSGLWDVLPEYLPALYSVCPGLTSLNLSYATVRMPDLVELLRRCSKLQKLWVMDLIEDKGLEAVASYCKELRELRVFPSEPDLDATNIPLTEQGLVFVSKGCRKLESVLYFCVQFTNAALFTIARKRPNLKCFRLCVIEPFAPDYKTNEPLDKGFKAIAEGCRDLRRLSVSGLLSDKAFKYIGKHAKKVRMLSIAFAGDSDLMLHHLLSGCESLKKLEIRDCPFGDTALLEHAAKLETMRSLWMSSCFVSFGACKLLSQKMPRLNVEVIDEHPPESRPESSPVERIYIYRTVAGPRMDTPEFVWTIHKNPENGVSHLAIK

**Supplementary methods**

***Molecular dynamics simulations***

We used the gaff2 force field the force field to obtain parameters for the compounds[72]. The charges for all the ligands were fit by antechamber[73] with the energy calculated by Gauusian16 B3LYP/def2TZVP basis[74]. Protonation of the protein-ligand complex was performed at pH 7.4. Molecular dynamics (MD) simulations were carried out using Amber22 with the ff19SB force field [75]. We solvated the protein-ligand complex in a cubic box using TIP3P water, which extends 10.0 Å away from the solute. Cut-off of non-bonded interactions was set to 10 Å. We added counter-ions to neutralize the systems. Each system was minimized for 10000 steps and then heated from 0 to 300 K in 300 ps with Langevin dynamics. Berendsen barostat was used to control the pressure at 1.0 atm. Production runs were 50-ns long in an NPT ensemble with a time step of 2 fs for 5 individual. The trajectories were recorded every 2 ps. We used the SHAKE algorithm to constrain the bonds involving hydrogen atoms[76].

***Binding free energy calculation of MMGBSA***

Calculate the binding free energy using MM-PB/GBSA (Molecular Mechanics Generalized Born Surface Area). Therefore, the equation:

$$\Delta G_{\text{bind}}=\Delta G_{\mathrm{complex}}-\Delta G_{protein1}-\Delta G_{protein2}=\Delta E_{\mathrm{vdw}}+\Delta E_{\mathrm{ele}}-T\Delta S+\Delta G_{\mathrm{polar}}+\Delta G_{nonpolar}$$
$\Delta\text{G}_{\text{complex}}$,$\Delta\text{G}_{\text{protein1}}$,$\Delta\text{G}_{\text{protein2}}$ represent the free energy of complex system, rprotein1, and protein2, respectively. $\Delta\text{E}_{\text{vdw}}$ and $\Delta\text{E}_{\text{ele}}$represent the changes in van der Waals and electrostatic energies during gas-phase binding, respectively. $\Delta\text{G}_{\text{polar}}$and$\Delta\text{G}_{\text{nonpolar}}$ represent the polar and nonpolar solvation energies, respectively.

***Binding free energy calculation of ASGBIE***

We used the alanine-scanning in combination with interaction entropy (AS-IE) method to calculate the binding free energy [77]. In this approach, it is assumed that the contribution of the mutated alanine to the binding free energy is negligible. Therefore, the contribution of a specific residue [78] to the total binding free energy is given by the energy difference when the wildtype residue X is mutated to A (alanine):

$\Delta\Delta\text{G}_{\text{bind}}^{\text{x}\to a}=\Delta\text{G}_{\text{bind}}^{a}-\Delta\text{G}_{\text{bind}}^{x}=\Delta\Delta\text{G}_{\text{gas}}^{\text{x}\to a}+\Delta\Delta\text{G}_{\text{sol}}^{\text{x}\to a}$ (a)

The gas-phase binding free energy and the solvation component are evaluated by the following scheme:

$\Delta\Delta\text{G}_{\text{gas}}^{\text{x}\to a}=\Delta\text{G}_{\text{gas}}^{a}-\Delta\text{G}_{\text{gas}}^{\text{x}}$ (b)

$\Delta\Delta\text{G}_{\text{sol}}^{\text{x}\to a}=\Delta\text{G}_{\text{sol}}^{a}-\Delta\text{G}_{\text{sol}}^{\text{x}}$ (c)

The gas-phase component of the binding free energy is $\Delta\text{G}_{\text{gas}}^{a}$ and $\Delta\text{G}_{\text{gas}}^{\text{x}}$, which represent the energy between the ligand L and alanine and the wildtype residue X. The enthalpy and entropy of gas-phase energy were calculated with the standard molecular mechanics and the IE method[79-82], respectively

$\Delta\text{G}_{\text{gas}}^{\text{x}}=\left\langle\text{E}_{\text{int}}^{\text{x}}\right\rangle-\text{T}\Delta\text{S}_{\mathrm{int}}^{x}=\left\langle\text{E}_{\text{int}}^{\text{x}}\right\rangle-K\text{Tln}\left\langle\text{e}^{\beta\Delta\text{E}_{\text{int}}^{\text{x}}}\right\rangle$ (e)

$\Delta\text{G}_{\text{gas}}^{\text{a}}=\left\langle\text{E}_{\text{int}}^{\text{a}}\right\rangle-K\text{Tln}\left\langle\text{e}^{\beta\Delta\text{E}_{\text{int}}^{\text{a}}}\right\rangle$ (f)

, where $\text{E}_{\text{int}}^{\text{a}}$ and $\text{E}_{\text{int}}^{\text{x}}$were the van der Waals and electrostatic interaction energies between the ligand and residue X and A, respectively. The exponential argument $\beta$ denotes $\frac{1}{\text{KT}}$, and $\Delta E_{\text{int}}^{\text{x/a}}$ is the deviation from average. We evaluated the exponential average$\text{E}_{\text{int}}^{\text{x/a}}$ by discrete time averaging,

$\left\langle\text{e}^{\beta\Delta\text{E}_{\text{int}}^{\text{x}}}\right\rangle=\frac{1}{\text{N}}\sum_{\text{i}=1}^{\text{N}} \text{e}^{\beta\Delta\text{E}_{\text{int}}^{\text{x}}(\text{t}_{\text{i}})}$ (g)

where N is the number of MD snapshots. Finally, Eq. (b) becomes

$\Delta\Delta\text{G}_{\text{gas}}^{\text{x}\to\text{a}}=\Delta\Delta E_{\text{gas}}^{\text{x}\to\text{a}}-T\Delta\Delta S_{gas}^{x\to a}=\left\langle\text{E}_{\text{int}}^{\text{a}}\right\rangle-\left\langle\text{E}_{\text{int}}^{\text{x}}\right\rangle+K\text{T[ln}\left\langle\text{e}^{\beta\Delta\text{E}_{\text{int}}^{\text{a}}}\right\rangle-\text{ln}\left\langle\text{e}^{\beta\Delta\text{E}_{\text{int}}^{\text{a}}}\right\rangle]$ (h)

The solvation energy in equation (c) was calculated using the OBC GBSA model (igb=2 in Amber22) [83-85] with a dielectric constant of 1, 3, and 10 for nonpolar, polar, and charged residues[86, 87].

The total protein-ligand binding free energy can be approximated by the summation[78, 88],

$\Delta G_{\text{bind}}=-\sum_{x} \Delta\Delta G_{\text{bind}}^{x\to a}$ (i)

where the summation is over residues that are within 5 Å of the ligand. A total of 100 snapshots from the last 5 ns of the MD trajectory were used to calculate the binding energy with the AS-IE methods.

**References**

[1] A. Shimabukuro-Vornhagen, P. Gödel, M. Subklewe, H.J. Stemmler, H.A. Schlößer, M. Schlaak, M. Kochanek, B. Böll, M.S. von Bergwelt-Baildon, Cytokine release syndrome, Journal for immunotherapy of cancer, 6 (2018) 56.

[2] X. Li, N. Gong, F. Tian, S. Zhang, Y. Zhang, Y. Wang, G. Qing, Y. Wang, F. Li, Y. Xu, L. Zhang, J. Wang, Q. Ni, Y. Gan, C. Gu, H. Jiang, X. Huang, X. Shi, T. Zhang, Y. Wu, X.-J. Liang, Suppression of cytokine release syndrome during CAR-T-cell therapy via a subcutaneously injected interleukin-6-adsorbing hydrogel, Nature Biomedical Engineering, 7 (2023) 1129-1141.

[3] X. Xiao, S. Huang, S. Chen, Y. Wang, Q. Sun, X. Xu, Y. Li, Mechanisms of cytokine release syndrome and neurotoxicity of CAR T-cell therapy and associated prevention and management strategies, Journal of experimental & clinical cancer research : CR, 40 (2021) 367.

[4] Y.G. Lee, H. Chu, Y. Lu, C.P. Leamon, M. Srinivasarao, K.S. Putt, P.S. Low, Regulation of CAR T cell-mediated cytokine release syndrome-like toxicity using low molecular weight adapters, Nature communications, 10 (2019) 2681.

[5] O. Castaneda-Puglianini, J.C. Chavez, Assessing and Management of Neurotoxicity After CAR-T Therapy in Diffuse Large B-Cell Lymphoma, Journal of blood medicine, 12 (2021) 775-783.

[6] B.D. Hunter, C.A. Jacobson, CAR T-Cell Associated Neurotoxicity: Mechanisms, Clinicopathologic Correlates, and Future Directions, Journal of the National Cancer Institute, 111 (2019) 646-654.

[7] P. Karschnia, J. Dietrich, Neurological complications of CAR T cell therapy for cancers, Nature Reviews Neurology, 21 (2025) 422-431.

[8] C.E. Graham, R. Velasco, A. Alarcon Tomas, O.P. Stewart, G. Dachy, F. Del Bufalo, M. Doglio, J.I. Henter, G. Ortí, Z. Peric, C. Roddie, N. van de Donk, M.J. Frigault, A. Ruggeri, F. Onida, I. Sánchez-Ortega, I. Yakoub-Agha, O. Penack, Non-ICANS neurological complications after CAR T-cell therapies: recommendations from the EBMT Practice Harmonisation and Guidelines Committee, The Lancet. Oncology, 26 (2025) e203-e213.

[9] C.L. Flugel, R.G. Majzner, G. Krenciute, G. Dotti, S.R. Riddell, D.L. Wagner, M. Abou-el-Enein, Overcoming on-target, off-tumour toxicity of CAR T cell therapy for solid tumours, Nature Reviews Clinical Oncology, 20 (2023) 49-62.

[10] L. Lu, M. Xie, B. Yang, W.B. Zhao, J. Cao, Enhancing the safety of CAR-T cell therapy: Synthetic genetic switch for spatiotemporal control, Sci Adv, 10 (2024) eadj6251.

[11] J. Chen, S. Qiu, W. Li, K. Wang, Y. Zhang, H. Yang, B. Liu, G. Li, L. Li, M. Chen, J. Lan, J. Niu, P. He, L. Cheng, G. Fan, X. Liu, X. Song, C. Xu, H. Wu, H. Wang, Tuning charge density of chimeric antigen receptor optimizes tonic signaling and CAR-T cell fitness, Cell Research, 33 (2023) 341-354.

[12] B.Z. Stanton, E.J. Chory, G.R. Crabtree, Chemically induced proximity in biology and medicine, 359 (2018) eaao5902.

[13] A. Fegan, B. White, J.C. Carlson, C.R. Wagner, Chemically controlled protein assembly: techniques and applications, Chemical reviews, 110 (2010) 3315-3336.

[14] T. Wang, S. Liu, Y. Ke, S. Ali, R. Wang, T. Hong, Z. Liu, G. Ma, T.-H. Lan, F. Wang, M.X. Zhu, Y. Huang, Y. Zhou, Repurposing salicylic acid as a versatile inducer of proximity, Nature Chemical Biology, (2025).

[15] J. Appelbaum, A.E. Price, K. Oda, J. Zhang, W.H. Leung, G. Tampella, D. Xia, P.P. So, S.K. Hilton, C. Evandy, S. Sarkar, U. Martin, A.R. Krostag, M. Leonardi, D.E. Zak, R. Logan, P. Lewis, S. Franke-Welch, N. Ngwenyama, M. Fitzgerald, N. Tulberg, S. Rawlings-Rhea, R.A. Gardner, K. Jones, A. Sanabria, W. Crago, J. Timmer, A. Hollands, B. Eckelman, S. Bilic, J. Woodworth, A. Lamble, P.D. Gregory, J. Jarjour, M. Pogson, J.A. Gustafson, A. Astrakhan, M.C. Jensen, Drug-regulated CD33-targeted CAR T cells control AML using clinically optimized rapamycin dosing, The Journal of clinical investigation, 134 (2024).

[16] C.Y. Wu, K.T. Roybal, E.M. Puchner, J. Onuffer, W.A. Lim, Remote control of therapeutic T cells through a small molecule-gated chimeric receptor, Science (New York, N.Y.), 350 (2015) aab4077.

[17] S.M. Lee, C.H. Kang, S.U. Choi, Y. Kim, J.Y. Hwang, H.G. Jeong, C.H. Park, A Chemical Switch System to Modulate Chimeric Antigen Receptor T Cell Activity through Proteolysis-Targeting Chimaera Technology, ACS synthetic biology, 9 (2020) 987-992.

[18] A.C. McCue, Z. Yao, B. Kuhlman, Advances in modular control of CAR-T therapy with adapter-mediated CARs, Advanced Drug Delivery Reviews, 187 (2022) 114358.

[19] L. Nikolaenko, C. Mulroney, P.A. Riedell, I.W. Flinn, J.C. Cruz, R. Vaidya, K. van Besien, M. Emde, V. Klyushnichenko, J. Strauss, E. Laborda, J. Li, M. Trikha, P.G. Schultz, T.S. Young, First in Human Study of an on/Off Switchable CAR-T Cell Platform Targeting CD19 for B Cell Malignancies (CLBR001 + SWI019), Blood, 138 (2021) 2822-2822.

[20] L. Lu, M. Xie, B. Yang, W.-b. Zhao, J. Cao, Enhancing the safety of CAR-T cell therapy: Synthetic genetic switch for spatiotemporal control, Science Advances, 10 eadj6251.

[21] N.T. Nguyen, K. Huang, H. Zeng, J. Jing, R. Wang, S. Fang, J. Chen, X. Liu, Z. Huang, M.J. You, A. Rao, Y. Huang, G. Han, Y. Zhou, Nano-optogenetic engineering of CAR T cells for precision immunotherapy with enhanced safety, Nature nanotechnology, 16 (2021) 1424-1434.

[22] L. Labanieh, R.G. Majzner, D. Klysz, E. Sotillo, C.J. Fisher, J.G. Vilches-Moure, K.Z.B. Pacheco, M. Malipatlolla, P. Xu, J.H. Hui, T. Murty, J. Theruvath, N. Mehta, S.A. Yamada-Hunter, E.W. Weber, S. Heitzeneder, K.R. Parker, A.T. Satpathy, H.Y. Chang, M.Z. Lin, J.R. Cochran, C.L. Mackall, Enhanced safety and efficacy of protease-regulated CAR-T cell receptors, Cell, 185 (2022) 1745-1763.e1722.

[23] H.-S. Li, N.M. Wong, E. Tague, J.T. Ngo, A.S. Khalil, W.W. Wong, High-performance multiplex drug-gated CAR circuits, Cancer Cell, 40 (2022) 1294-1305.e1294.

[24] A. Praznik, T. Fink, N. Franko, J. Lonzarić, M. Benčina, N. Jerala, T. Plaper, S. Roškar, R. Jerala, Regulation of protein secretion through chemical regulation of endoplasmic reticulum retention signal cleavage, Nature communications, 13 (2022) 1323.

[25] C.U. Zajc, M. Dobersberger, I. Schaffner, G. Mlynek, D. Pühringer, B. Salzer, K. Djinović-Carugo, P. Steinberger, A. De Sousa Linhares, N.J. Yang, C. Obinger, W. Holter, M.W. Traxlmayr, M. Lehner, A conformation-specific ON-switch for controlling CAR T cells with an orally available drug, Proceedings of the National Academy of Sciences, 117 (2020) 14926-14935.

[26] G.M.P. Giordano Attianese, S. Shui, E. Cribioli, M. Triboulet, L. Scheller, M. Hafezi, P. Reichenbach, P. Gainza, S. Georgeon, B.E. Correia, M. Irving, Dual ON/OFF-switch chimeric antigen receptor controlled by two clinically approved drugs, Proceedings of the National Academy of Sciences of the United States of America, 121 (2024) e2405085121.

[27] M. Jan, I. Scarfò, R.C. Larson, A. Walker, A. Schmidts, A.A. Guirguis, J.A. Gasser, M. Słabicki, A.A. Bouffard, A.P. Castano, M.C. Kann, M.L. Cabral, A. Tepper, D.E. Grinshpun, A.S. Sperling, T. Kyung, Q.L. Sievers, M.E. Birnbaum, M.V. Maus, B.L. Ebert, Reversible ON- and OFF-switch chimeric antigen receptors controlled by lenalidomide, Science translational medicine, 13 (2021).

[28] S. Li, L. Shi, L. Zhao, Q. Guo, J. Li, Z.-l. Liu, Z. Guo, Y.J. Cao, Split-design approach enhances the therapeutic efficacy of ligand-based CAR-T cells against multiple B-cell malignancies, Nature communications, 15 (2024) 9751.

[29] D.L. Wagner, E. Fritsche, M.A. Pulsipher, N. Ahmed, M. Hamieh, M. Hegde, M. Ruella, B. Savoldo, N.N. Shah, C.J. Turtle, A.S. Wayne, M. Abou-el-Enein, Immunogenicity of CAR T cells in cancer therapy, Nature Reviews Clinical Oncology, 18 (2021) 379-393.

[30] X. Chen, S. Wang, Y. Chen, H. Xin, S. Zhang, D. Wu, Y. Xue, M. Zha, H. Li, K. Li, Z. Gu, W. Wei, Y. Ping, Non-invasive activation of intratumoural gene editing for improved adoptive T-cell therapy in solid tumours, Nature nanotechnology, 18 (2023) 933-944.

[31] X. Wang, R. Fan, M. Mu, L. Zhou, B. Zou, A. Tong, G. Guo, Harnessing nanoengineered CAR-T cell strategies to advance solid tumor immunotherapy, Trends in Cell Biology.

[32] Z. Zhao, Q. Li, C. Qu, Z. Jiang, G. Jia, G. Lan, Y. Luan, A collagenase nanogel backpack improves CAR-T cell therapy outcomes in pancreatic cancer, Nature nanotechnology, (2025).

[33] C. Amatya, M.A. Pegues, N. Lam, D. Vanasse, C. Geldres, S. Choi, S.M. Hewitt, S.A. Feldman, J.N. Kochenderfer, Development of CAR T Cells Expressing a Suicide Gene Plus a Chimeric Antigen Receptor Targeting Signaling Lymphocytic-Activation Molecule F7, Molecular therapy : the journal of the American Society of Gene Therapy, 29 (2021) 702-717.

[34] C.A. Wills, D. Drago, R.G. Pietrusko, Clinical holds for cell and gene therapy trials: Risks, impact, and lessons learned, Molecular therapy. Methods & clinical development, 31 (2023) 101125.

[35] M.N. Stein, E.E. Dumbrava, B.A. Teply, U.S. Gergis, M.E. Guiterrez, R. Reshef, S.K. Subudhi, C.F. Jacquemont, J.H. Senesac, J.H. Bayle, C.D. Scripture, M.S. Chatwal, M.A. Bilen, W.M. Stadler, C.R. Becerra, PSCA-targeted BPX-601 CAR T cells with pharmacological activation by rimiducid in metastatic pancreatic and prostate cancer: a phase 1 dose escalation trial, Nature communications, 15 (2024) 10743.

[36] M.R.A. Figueiredo, L.C. Strader, Intrinsic and extrinsic regulators of Aux/IAA protein degradation dynamics, Trends in biochemical sciences, 47 (2022) 865-874.

[37] W.M. Gray, S. Kepinski, D. Rouse, O. Leyser, M. Estelle, Auxin regulates SCFTIR1-dependent degradation of AUX/IAA proteins, Nature, 414 (2001) 271-276.

[38] K.M. Sathyan, B.D. McKenna, W.D. Anderson, F.M. Duarte, L. Core, M.J. Guertin, An improved auxin-inducible degron system preserves native protein levels and enables rapid and specific protein depletion, Genes & development, 33 (2019) 1441-1455.

[39] K. Nishimura, T. Fukagawa, H. Takisawa, T. Kakimoto, M. Kanemaki, An auxin-based degron system for the rapid depletion of proteins in nonplant cells, Nature methods, 6 (2009) 917-922.

[40] A. Yesbolatova, Y. Saito, N. Kitamoto, H. Makino-Itou, R. Ajima, R. Nakano, H. Nakaoka, K. Fukui, K. Gamo, Y. Tominari, H. Takeuchi, Y. Saga, K.I. Hayashi, M.T. Kanemaki, The auxin-inducible degron 2 technology provides sharp degradation control in yeast, mammalian cells, and mice, Nature communications, 11 (2020) 5701.

[41] J. López-Bucio, Dietary auxin may help patients to fight cancer, Trends in plant science, 30 (2025) 134-136.

[42] L. Sethi, K. Kumari, N. Dey, Engineering of Plants for Efficient Production of Therapeutics, Molecular Biotechnology, 63 (2021) 1125-1137.

[43] M. Laskowski, Teaching resources. Model of the TIR1 pathway for auxin-mediated gene expression, Science's STKE : signal transduction knowledge environment, 2006 (2006) tr1.

[44] X. Tan, L.I.A. Calderon-Villalobos, M. Sharon, C. Zheng, C.V. Robinson, M. Estelle, N. Zheng, Mechanism of auxin perception by the TIR1 ubiquitin ligase, Nature, 446 (2007) 640-645.

[45] N.B.C. Serre, D. Kralík, P. Yun, Z. Slouka, S. Shabala, M. Fendrych, AFB1 controls rapid auxin signalling through membrane depolarization in Arabidopsis thaliana root, Nature Plants, 7 (2021) 1229-1238.

[46] L. Qi, M. Kwiatkowski, H. Chen, L. Hoermayer, S. Sinclair, M. Zou, C.I. del Genio, M.F. Kubeš, R. Napier, K. Jaworski, J. Friml, Adenylate cyclase activity of TIR1/AFB auxin receptors in plants, Nature, 611 (2022) 133-138.

[47] L.I.A. Calderón Villalobos, S. Lee, C. De Oliveira, A. Ivetac, W. Brandt, L. Armitage, L.B. Sheard, X. Tan, G. Parry, H. Mao, N. Zheng, R. Napier, S. Kepinski, M. Estelle, A combinatorial TIR1/AFB–Aux/IAA co-receptor system for differential sensing of auxin, Nature Chemical Biology, 8 (2012) 477-485.

[48] F. Cui, S. Wu, W. Sun, G. Coaker, B. Kunkel, P. He, L. Shan, The Pseudomonas syringae Type III Effector AvrRpt2 Promotes Pathogen Virulence via Stimulating Arabidopsis Auxin/Indole Acetic Acid Protein Turnover      Plant Physiology, 162 (2013) 1018-1029.

[49] S. Li, Y. Wang, M. van der Stoel, X. Zhou, S. Madhusudan, K. Kanerva, V.D. Nguyen, N. Eskici, V.M. Olkkonen, Y. Zhou, T. Raivio, E. Ikonen, HiHo-AID2: boosting homozygous knock-in efficiency enables robust generation of human auxin-inducible degron cells, Genome Biology, 25 (2024) 58.

[50] S. Li, X. Prasanna, V.T. Salo, I. Vattulainen, E. Ikonen, An efficient auxin-inducible degron system with low basal degradation in human cells, Nature methods, 16 (2019) 866-869.

[51] J. Hyle, M.N. Djekidel, J. Williams, S. Wright, Y. Shao, B. Xu, C. Li, Auxin-inducible degron 2 system deciphers functions of CTCF domains in transcriptional regulation, Genome Biology, 24 (2023) 14.

[52] M. Capece, A. Tessari, J. Mills, G.L.R. Vinciguerra, D. Louke, C. Lin, B.K. McElwain, W.O. Miles, V. Coppola, A.E. Davies, D. Palmieri, C.M. Croce, A novel auxin-inducible degron system for rapid, cell cycle-specific targeted proteolysis, Cell Death & Differentiation, 30 (2023) 2078-2091.

[53] T. Negishi, S. Kitagawa, N. Horii, Y. Tanaka, N. Haruta, A. Sugimoto, H. Sawa, K.-i. Hayashi, M. Harata, M.T. Kanemaki, The auxin-inducible degron 2 (AID2) system enables controlled protein knockdown during embryogenesis and development in Caenorhabditis elegans, Genetics, 220 (2021).

[54] S. Li, R. Zhao, D. Zheng, L. Qin, Y. Cui, Y. Li, Z. Jiang, M. Zhong, J. Shi, M. Li, X. Wang, Z. Tang, Q. Wu, Y. Long, D. Hu, S. Wang, Y. Yao, S. Liu, L.H. Yang, Z. Zhang, Q. Tang, P. Liu, Y. Li, P. Li, DAP10 integration in CAR-T cells enhances the killing of heterogeneous tumors by harnessing endogenous NKG2D, Molecular therapy oncolytics, 26 (2022) 15-26.

[55] T. Nagai, K. Ibata, E.S. Park, M. Kubota, K. Mikoshiba, A. Miyawaki, A variant of yellow fluorescent protein with fast and efficient maturation for cell-biological applications, Nature Biotechnology, 20 (2002) 87-90.

[56] D.S. Bindels, L. Haarbosch, L. van Weeren, M. Postma, K.E. Wiese, M. Mastop, S. Aumonier, G. Gotthard, A. Royant, M.A. Hink, T.W. Gadella, Jr., mScarlet: a bright monomeric red fluorescent protein for cellular imaging, Nature methods, 14 (2017) 53-56.

[57] J. Lim, M. Petersen, M. Bunz, C. Simon, M. Schindler, Flow cytometry based-FRET: basics, novel developments and future perspectives, Cellular and molecular life sciences : CMLS, 79 (2022) 217.

[58] S. Vogel, A Practical Guide to Quantitative FLIM/FRET, J Biomol Tech. 2020 Aug;31(Suppl):S32.

[59] J. Tintelnot, Y. Xu, T.R. Lesker, M. Schönlein, L. Konczalla, A.D. Giannou, P. Pelczar, D. Kylies, V.G. Puelles, A.A. Bielecka, M. Peschka, F. Cortesi, K. Riecken, M. Jung, L. Amend, T.S. Bröring, M. Trajkovic-Arsic, J.T. Siveke, T. Renné, D. Zhang, S. Boeck, T. Strowig, F.G. Uzunoglu, C. Güngör, A. Stein, J.R. Izbicki, C. Bokemeyer, M. Sinn, A.C. Kimmelman, S. Huber, N. Gagliani, Microbiota-derived 3-IAA influences chemotherapy efficacy in pancreatic cancer, Nature, 615 (2023) 168-174.

[60] N. Uchida, K. Takahashi, R. Iwasaki, R. Yamada, M. Yoshimura, T.A. Endo, S. Kimura, H. Zhang, M. Nomoto, Y. Tada, T. Kinoshita, K. Itami, S. Hagihara, K.U. Torii, Chemical hijacking of auxin signaling with an engineered auxin-TIR1 pair, Nat Chem Biol, 14 (2018) 299-305.

[61] S. Rafiq, C.S. Hackett, R.J. Brentjens, Engineering strategies to overcome the current roadblocks in CAR T cell therapy, Nature reviews. Clinical oncology, 17 (2020) 147-167.

[62] M. Elmarasi, I. Elkonaissi, A.A. Elsabagh, E. Elsayed, A. Elsayed, B. Elsayed, I. Elmakaty, M. Yassin, CAR-T cell therapy: Efficacy in management of cancers, adverse effects, dose-limiting toxicities and long-term follow up, International Immunopharmacology, 135 (2024) 112312.

[63] V. Shakhnovich, B. Meibohm, A. Rosenberg, A.M. Kierzek, R. Hasenkamp, R.S. Funk, C.J. Thalhauser, P.H. van der Graaf, Y.C. Wang, L. Hamuro, Immunogenicity in Clinical Practice and Drug Development: When is it Significant?, Clinical and translational science, 13 (2020) 219-223.

[64] D. Zhou, L.A. Byers, B. Sable, M.D. Smit, N.H. Sadraei, S. Dutta, V.V. Upreti, Clinical Pharmacology Profile of AMG 119, the First Chimeric Antigen Receptor T (CAR-T) Cell Therapy Targeting Delta-Like Ligand 3 (DLL3), in Patients with Relapsed/Refractory Small Cell Lung Cancer (SCLC), Journal of clinical pharmacology, 64 (2024) 362-370.

[65] J.H. Bayle, M.T. Duong, A. Lu, E. Morschl, M.R. Collinson-Pautz, K.L. Sharp, S. Szymanski, M.E. Brandt, K.M. Slawin, S. Toler, E. Yvon, A. Foster, D.M. Spencer, Dual-Switch CAR-T Cells: Orthogonal Molecular Switches to Control Activation and Elimination of CAR-T Cells to Target CD123+ Cancer, Blood, 130 (2017) 3184.

[66] M. Bachmann, The UniCAR system: A modular CAR T cell approach to improve the safety of CAR T cells, Immunology letters, 211 (2019) 13-22.

[67] J.H. Cho, J.J. Collins, W.W. Wong, Universal Chimeric Antigen Receptors for Multiplexed and Logical Control of T Cell Responses, Cell, 173 (2018) 1426-1438.e1411.

[68] O. Roth, S. Yechezkel, O. Serero, A. Eliyahu, I. Vints, P. Tzeela, A. Carignano, D.P. Janacek, V. Peters, A. Kessel, V. Dwivedi, M. Carmeli-Weissberg, F. Shaya, A. Faigenboim-Doron, K.L. Ung, B.P. Pedersen, J. Riov, E. Klavins, C. Dawid, U.Z. Hammes, N. Ben-Tal, R. Napier, E. Sadot, R. Weinstain, Slow release of a synthetic auxin induces formation of adventitious roots in recalcitrant woody plants, Nat Biotechnol, 42 (2024) 1705-1716.

[69] B. Xie, Z. Li, J. Zhou, W. Wang, Current Status and Perspectives of Dual-Targeting Chimeric Antigen Receptor T-Cell Therapy for the Treatment of Hematological Malignancies, Cancers, 14 (2022).

[70] S. Ouladan, E. Orouji, Chimeric Antigen Receptor-T Cells in Colorectal Cancer: Pioneering New Avenues in Solid Tumor Immunotherapy, Journal of clinical oncology : official journal of the American Society of Clinical Oncology, 43 (2025) 994-1005.

[71] M. Verma, K. Obergfell, S. Topp, V. Panier, J. Wu, The next-generation CAR-T therapy landscape, Nature reviews. Drug discovery, 22 (2023) 776-777.

[72] J. Wang, R.M. Wolf, J.W. Caldwell, P.A. Kollman, D.A. Case, Development and testing of a general amber force field, J Comput Chem, 25 (2004) 1157-1174.

[73] R. Salomon-Ferrer, D.A. Case, R.C. Walker, An overview of the Amber biomolecular simulation package, Wiley Interdisciplinary Reviews: Computational Molecular Science, 3 (2013) 198-210.

[74] M.J.F.a.G.W.T.a.H.B.S.a.G.E.S.a.M.A.R.a.J.R.C.a. G., Gaussian 16 Rev. C.01, 2016.

[75] J.A. Maier, C. Martinez, K. Kasavajhala, L. Wickstrom, K.E. Hauser, C. Simmerling, ff14SB: Improving the Accuracy of Protein Side Chain and Backbone Parameters from ff99SB, J Chem Theory Comput, 11 (2015) 3696-3713.

[76] H.C. Andersen, Rattle: A “velocity”version of the shake algorithm for molecular dynamics calculations, J. Comput. Phys., 52 (1983) 24-34.

[77] Y. Yan, M. Yang, C.G. Ji, J.Z.H. Zhang, Interaction Entropy for Computational Alanine Scanning, J Chem Inf Model, 57 (2017) 1112-1122.

[78] Y. Zhou, X. Liu, Y. Zhang, L. Peng, J.Z.H. Zhang, Residue-specific free energy analysis in ligand bindings to JAK2, Molecular Physics, 116 (2018) 2633-2641.

[79] L. Duan, X. Liu, J.Z. Zhang, Interaction Entropy: A New Paradigm for Highly Efficient and Reliable Computation of Protein-Ligand Binding Free Energy, J Am Chem Soc, 138 (2016) 5722-5728.

[80] L.Q. Qiu, Y.N. Yan, Z.X. Sun, J.N. Song, J.Z.H. Zhang, Interaction entropy for computational alanine scanning in protein-protein binding, Wires Comput Mol Sci, 8 (2018).

[81] J. Song, L. Qiu, J.Z.H. Zhang, An efficient method for computing excess free energy of liquid, Science China Chemistry, 61 (2017) 135-140.

[82] I.Y. Ben-Shalom, S. Pfeiffer-Marek, K.H. Baringhaus, H. Gohlke, Efficient Approximation of Ligand Rotational and Translational Entropy Changes upon Binding for Use in MM-PBSA Calculations, J Chem Inf Model, 57 (2017) 170-189.

[83] S. Genheden, U. Ryde, The MM/PBSA and MM/GBSA methods to estimate ligand-binding affinities, Expert Opin Drug Discov, 10 (2015) 449-461.

[84] I. Massova, P.A. Kollman, Combined molecular mechanical and continuμM solvent approach (MM-PBSA/GBSA) to predict ligand binding, Perspectives in Drug Discovery and Design, 18 (2000) 113-135.

[85] A. Onufriev, D. Bashford, D.A. Case, Exploring protein native states and large-scale conformational changes with a modified generalized born model, Proteins, 55 (2004) 383-394.

[86] M. Petukh, M. Li, E. Alexov, Predicting Binding Free Energy Change Caused by Point Mutations with Knowledge-Modified MM/PBSA Method, PLoS Comput Biol, 11 (2015) e1004276.

[87] T. Hou, J. Wang, Y. Li, W. Wang, Assessing the performance of the MM/PBSA and MM/GBSA methods. 1. The accuracy of binding free energy calculations based on molecular dynamics simulations, J Chem Inf Model, 51 (2011) 69-82.

[88] X. Liu, L. Peng, Y. Zhou, Y. Zhang, J.Z.H. Zhang, Computational Alanine Scanning with Interaction Entropy for Protein-Ligand Binding Free Energies, J Chem Theory Comput, 14 (2018) 1772-1780.
